# Reshaping of the fecal proteome and metaproteome in obese patients 2 years after bariatric surgery

**DOI:** 10.1128/msystems.01764-25

**Published:** 2026-06-09

**Authors:** Alessandro Tanca, Laura De Diego, Maria Antonietta Deledda, Flavio De Maio, Cristian Boru, Mario Musella, Marco Raffaelli, Gianfranco Silecchia, Giovanni Delogu, Sergio Uzzau

**Affiliations:** 1 Department of Biomedical Sciences, University of Sassari 390773, Sassari, Italy; 2 Unit of Microbiology and Virology, University Hospital of Sassari 9312, Sassari, Italy; 3 Department of Laboratory and Hematology Sciences, Fondazione Policlinico Universitario A. Gemelli IRCCS 18654, Rome, Italy; 4 Università Cattolica del Sacro Cuore 96983 , Rome, Italy; 5 Department of Medical-Surgical Sciences and Translational Medicine, Faculty of Medicine and Psychology, University "La Sapienza" of Rome 9311, Rome, Italy; 6 Department of Advanced Biomedical Sciences, University of Naples "Federico II" 507764, Naples, Italy; 7 Division of Endocrine and Metabolic Surgery, Fondazione Policlinico A. Gemelli IRCCS, Università Cattolica del Sacro Cuore 96983 , Rome, Italy; 8 Department of Medicine and Surgery, University of Parma 478519, Parma, Italy; 9 Unit of Microbiology, University Hospital of Parma 18630 , Parma, Italy; Cleveland Clinic, Cleveland, Ohio, USA

**Keywords:** bariatric surgery, gut microbiota, mass spectrometry, metabolism, metaproteomics, obesity, proteomics

## Abstract

Bariatric surgery provides effective and durable weight loss in patients with severe obesity. Both the surgical procedure and postoperative management, including dietary and behavioral changes, are known to induce long-term alterations in gut microbiota composition. However, their impact on microbial and host proteome profiles remains poorly understood. In this prospective, multicenter cohort study, we compared the fecal metaproteome profile of 45 patients with severe obesity at preoperative baseline (T0) and 2 years after surgery (T1). Patients were randomized to receive either Roux-en-Y gastric bypass or one anastomosis gastric bypass. At T1, the relative abundance of the bacterial genera *Akkermansia*, *Anaerotignum*, *Desulfovibrio*, *Streptococcus*, and *Veillonella* significantly increased, whereas that of *Faecalibacterium* and *Romboutsia* decreased. Furthermore, we observed a significant increase in the relative abundance of microbial enzymes involved in glycolysis, short-chain fatty acid biosynthesis, amino acid metabolism, and cofactor/vitamin biosynthesis, alongside various outer membrane proteins encoded by *Enterobacterales*. Conversely, several proteins involved in oxidative phosphorylation and sporulation were reduced. Moreover, 74 human proteins, primarily associated with immune functions, showed a significant increase in relative abundance at T1, while numerous digestive enzymes displayed a downward trend. Our data reveal that a distinct reshaping of the fecal proteome and metaproteome persists 2 years after bariatric surgery. Future studies are needed to elucidate the mechanistic aspects of host-microbiota interaction and to identify associations with long-term clinical outcomes.

**IMPORTANCE:**

Bariatric surgery is widely recognized as the most effective and durable intervention for severe obesity; however, its long-term molecular effects on gut microbiota-host interactions remain poorly understood. By applying shotgun metaproteomics to fecal samples collected before and 2 years after surgery, our study provides novel insights into the functional consequences of bariatric bypass procedures. We demonstrate sustained alterations in both microbial and host protein profiles, including metabolic enzymes, outer membrane proteins, and immune-related factors, revealing a long-lasting remodeling of gut ecosystem functions. These findings underscore the value of metaproteomics in uncovering molecular mechanisms underlying bariatric surgery outcomes and may ultimately guide the development of microbiome- or host-targeted strategies to optimize therapy and long-term patient care.

## INTRODUCTION

Bariatric surgery is a highly effective therapeutic option for patients affected by severe obesity (body mass index [BMI] >35 kg/m^2^), achieving successful outcomes in terms of weight loss and reduction of adiposity. The term bariatric/metabolic surgery (BMS) is now preferred, as it emphasizes the powerful effect of these procedures on improving metabolic control in diseases such as type 2 diabetes (1). Two of the main BMS techniques, Roux-en-Y gastric bypass (RYGB) and one anastomosis gastric bypass (OAGB), entail the surgical exclusion of a segment of the small intestine from the nutrient stream, thereby reducing the absorptive surface area and contributing to reduced nutrient absorption. This intentional hypoabsorptive component contributes to weight loss and metabolic improvements (2). Given the anatomical and physiological changes that occur after gastric bypass surgery, patients are at increased risk of micronutrient deficiencies. They therefore require management by a dietitian-nutritionist to ensure proper nutritional assessment, postoperative monitoring, and appropriate supplementation (3). However, the long-term success of these procedures is also influenced by additional factors, still not fully understood, that modulate central and peripheral appetite regulation as well as metabolic control (4).

Such anatomical and physiological modifications of the gastrointestinal tract are expected to significantly impact the composition and functionality of the gut microbiota (GM). While numerous clinical trials have consistently reported significant alterations to the GM composition in obese patients undergoing RYGB, OAGB, and vertical sleeve gastrectomy (VSG) (5–7), the first proof of a specific contribution of the GM to reduced weight and adiposity after BMS in experimental animal models was provided by Liou and coworkers in 2013 (8). Hence, a key role of the GM in the long-term success of BMS has been postulated, also given the potential impact of microbial metabolites on the complex network of receptors, hormones, and enzymatic functions involved in physiological systems, namely the gut-brain axis and the gut-liver axis, that contribute to appetite, energy expenditure, and metabolism (9). Three months post-intervention, shotgun sequencing analysis of human fecal samples showed more extensive changes in the relative abundance of several bacterial taxa after RYGB than after VSG, with common variations including a relative increase in *Streptococcus*, *Escherichia*, and *Anaerotruncus* and a relative decrease in *Faecalibacterium*, *Dorea,* and *Anaerostipes* (10, 11). GM changes after RYGB and VSG were also compared at 3, 9, and 12 months post-intervention, with RYGB leading to numerous changes at the phylum and genus levels associated with significant weight loss (11, 12). In contrast, only a small number of changes in the relative abundance of microbial genera were observed after VSG, with no changes in alpha-diversity (12). Notably, an increased abundance of the genera *Klebsiella*, *Escherichia*, *Streptococcus*, and *Veillonella* was observed at both short- and long-term follow-ups following RYGB (13). The more pronounced effects of RYGB compared to VSG may be attributed to the absence of small intestinal bypass in the latter, resulting in reduced hypoabsorption and, consequently, less weight loss. This differential outcome was also evident when comparing RYGB with laparoscopic adjustable gastric banding (LAGB) (14).

While of greatest interest and significance, almost all the current evidence examining the GM alterations in BMS clinical trials is based on DNA sequencing data, which lack information on the actual functional remodeling of the GM. A pioneering investigation into functional variations occurring in the human GM following BMS has been provided by Sanchez-Carrillo and colleagues (15). Using a metaproteomic approach, these authors investigated the changes in the relative abundance of microbial functions in the pooled fecal material of 40 severely obese individuals at baseline and after a calorie-restricted diet followed by RYGB or VSG. The study reported changes in the abundance of several GM functions related to energy production and redox balance 3 months after BMS. Their valuable, yet preliminary, results highlight how metaproteomics is beginning to advance the understanding of biological processes involving the GM by enabling the analysis of the longitudinal dynamics of microbial functions within the microbial community (16).

To date, a single DNA sequencing-based study has directly compared GM alterations 2 years after RYGB and OAGB, reporting only minor differences between the two procedures. These findings likely reflect the similar physiological impact of small intestinal bypass shared by both surgical techniques (17). Here, we extend this analysis within the same patient cohort by characterizing not only structural changes in the GM but also its functional dynamics. Through metaproteomic profiling of stool samples, we further assess variations in the host fecal proteome, thereby providing an integrated view of host-microbe post-surgery interactions.

## MATERIALS AND METHODS

### Patients and samples

Forty-five patients affected by severe obesity, candidates to laparoscopic BMS between May 2018 and January 2020 in three Italian, academic, high-volume centers, were selected from a multicenter, prospective, cohort study described elsewhere (17). Inclusion and exclusion criteria, study design, approvals, and registration on clinicaltrials.gov (Unique Protocol ID: NCT03412149) were previously reported (18). Patients were randomized between Roux-en-Y gastric bypass (RYGB) and one anastomosis gastric bypass (OAGB/MGB) groups. Detailed patients’ characteristics are provided in Table S1. Following BMS, management by a dietitian-nutritionist was performed. The focus of dietary counseling was the adaptation of patients’ eating behavior to the surgical procedure and the general qualitative aspects of a healthy, nutrient-dense diet (19). A standard, supplementary long-term regimen was recommended to all operated patients, including vitamins B1, B9, B12, folate, D, and iron (3).

Fresh stool samples were collected before (T0) and after (T1) surgical intervention from the 45 patients included in the study and stored at −80°C until processing. The study was conducted in accordance with the principles of the CONSORT 2010 statement (20).

### Protein extraction and digestion

Protein extraction from fecal samples was performed as follows. After thawing, approximately 200 mg of material was collected from each fecal sample. After the addition of a 5-mm steel bead (Qiagen, Hilden, Germany) and extraction buffer (2% SDS, 100 mM DTT, 20 mM Tris-HCl [pH 8. 5], with 2 μL of buffer added per mg of fecal pellet), the samples were incubated at 95°C for 10 min at 500 rpm using a Fisherbrand Isotemp Cooling Shake Touch Shaker (Thermo Fisher Scientific, Waltham, USA), homogenized at 2 m/s for 10 min using a Fisherbrand Bead Mill 24 Homogenizer (Thermo Fisher Scientific), and centrifuged at 16,000 × *g* for 10 min. At the end, the supernatants (protein extracts) were collected and analyzed by SDS-PAGE to estimate the protein concentration. The protein extracts (40–200 μL, depending on the estimated protein concentration) were then concentrated and purified by protein precipitation with trichloroacetic acid (TCA) and acetone. Specifically, an equal volume of 20% TCA solution was added to each protein extract, mixed by inversion, and incubated for 30 min on ice. After centrifugation at 16,000 × *g* for 15 min at 4°C, the TCA supernatants were removed, and 250 μL of cold acetone was added. The samples were then vortexed for 1 min, incubated for 2 h at −20°C, and centrifuged at 16,000 × *g* for 10 min at 4°C. After removal of the acetone supernatant, protein pellets were resuspended in 100 μL of solubilization buffer (8 M urea, 20 mM DTT, and 20 mM Tris-HCl, pH 8.5).

Proteins were then processed according to a modified filter-aided sample preparation protocol (21, 22). Accordingly, 20–50 µL of the resuspended proteins were loaded onto an Amicon Ultra-0.5 filtration device (30 kDa cutoff; Merck, Darmstadt, Germany) and centrifuged at 14,000 × *g* for 15 min. Then, 200 μL of solubilization buffer, 100 μL of 50 mM iodoacetamide in solubilization buffer, 100 μL of solubilization buffer (twice), and 100 μL of 50 mM ammonium bicarbonate were added sequentially to the filters, with each step followed by centrifugation at 14,000 × *g* for 10 min. Finally, proteins were digested with trypsin (1 μg per sample, dissolved in 50 mM ammonium bicarbonate solution) overnight at 37°C. After centrifugation at 14,000 × *g* for 15 min, the first eluate was collected. Then, 100 μL of elution solution (20% acetonitrile and 0.2% formic acid) was added to each filter, and after centrifugation at 14,000 × *g* for 15 min, a second eluate was collected and mixed with the first. Finally, the peptide mixtures were concentrated using a Concentrator Plus (Eppendorf, Hamburg, Germany) and stored at −20°C until liquid chromatography-tandem mass spectrometry (LC-MS/MS) analysis.

### LC-MS/MS analysis

LC-MS/MS analysis was conducted in the Proteomics Laboratory at Porto Conte Ricerche Srl (Alghero, Italy) using an LTQ Orbitrap Velos mass spectrometer (Thermo Fisher Scientific), equipped with an EASY-spray ion source and coupled to an UltiMate 3000 RSLCnano LC system (Thermo Fisher Scientific). Peptide mixtures (4 μg per run) were first loaded, concentrated, and desalted on a trapping pre-column (Acclaim PepMap C18, 75 μm × 2 cm nanoViper, 3 μm, 100 Å, Thermo Fisher Scientific) with a mobile phase of 0.05% trifluoroacetic acid in 2% acetonitrile at a flow rate of 5 μL/min. The trap loading duration was set to 3.1 min. Peptide separation was then carried out using a C18 EASY-spray column (PepMap RSLC C18, 75 μm × 50 cm, 2 μm, 100 Å, Thermo Fisher Scientific) at 35°C and a flow rate of 250 nL/min over a 247-min gradient. The gradient consisted of two phases: 2.5%–37.5% eluent B (0.1% formic acid in 80% acetonitrile) in eluent A (0.1% formic acid) for 242 min, followed by 37.5%–99% eluent B for 5 min. Samples were analyzed in a randomized order. The mass spectrometer was set up in data-dependent acquisition (DDA) mode as described previously (23).

### Bioinformatic analysis

Peptide identification and quantification were carried out using Proteome Discoverer (v.2.5; Thermo Fisher Scientific), as described previously (24). Sequest-HT was used as the search engine with the following search parameters: precursor mass range, 350–5,000 Da; minimum peak count, 6; S/N threshold, 2; enzyme, trypsin (full); maximum missed cleavage sites, 2; peptide length range, 5–50 amino acids; precursor mass tolerance, 10 ppm; fragment mass tolerance, 0.02 Da; static modification, cysteine carbamidomethylation; dynamic modification, methionine oxidation. Searches were conducted against a combination of two sequence databases, namely, a collection of human gut metagenomes (available at https://ftp.cngb.org/pub/SciRAID/Microbiome/humanGut_9.9M/GeneCatalog/IGC.pep.gz) (25) and the *Homo sapiens* reference proteome retrieved from UniProtKB/Swiss-Prot (release 2021_04). Peptides were categorized as “microbial” or “human” when belonging to the first or second database, respectively; peptides that matched both databases were categorized as “ambiguous.” Percolator was employed for peptide validation, setting the false-discovery rate (FDR) threshold to 1% at the PSM, peptide, and protein levels. Offline mass recalibration and label-free MS1 quantitation were performed using Spectrum Files RC and Minora Feature Detector nodes, respectively. Optimal settings for retention time and mass tolerance windows were calculated by Minora based on the mass accuracy and retention time variance distributions. A consensus feature list was defined based on the outputs of Feature Mapper and Precursor Ions Quantifier nodes. The MS1 signals of all peptides significantly matching with at least one MS2 spectrum from at least one sample were mapped across runs and quantified by calculating the integrated area of the chromatographic peak.

Unipept Desktop (v.2.0.0) was used to carry out peptide taxonomic annotation (26), selecting the three available options (“equate I and L,” “filter duplicate peptides,” and “advanced missed cleavage handling”). Protein sequences were subjected to functional annotation using the eggNOG-mapper web application (v.2.1.12, available at http://eggnog-mapper.embl.de/ at the time of the analysis) (27), keeping default parameters and then choosing Cluster of Orthologous Groups (COG) and Kyoto Encyclopedia of Genes and Genomes (KEGG) orthology (KO) as the main functional classifications (28, 29). Meta4P (v.1.5.5) was used to parse identification, quantification, and annotation data and generate aggregated abundance tables (30). Microbial and host peptides were processed in separate analyses. Microbial peptide abundances were normalized across samples based on the total microbial peptide abundance measured in each sample; for peptides mapping to multiple proteins, the functional annotations of all the mapped proteins were considered. The abundances of bacterial KEGG KO functions were calculated as follows: (i) regardless of the taxonomic annotation (i.e., generically assigned to “bacteria”) and (ii) based on the specific annotation at various taxonomic levels. The abundance of any given microbial taxon, function, or taxon-specific function was estimated by summing the normalized abundances of all peptides annotated with that feature(s). For example, the abundance of an order-specific KO function (e.g., enolase encoded by *Bacteroidales*) was calculated by summing the abundances of all microbial peptides having enolase (K01689) as KO functional annotation and *Bacteroidales* as taxonomic annotation at the order level. Host peptides were grouped into master proteins by Proteome Discoverer’s protein grouping algorithm, according to the principle of maximum parsimony. Master protein abundances were normalized based on the total host protein abundance measured in each sample. Ambiguous peptides were excluded from the final analysis.

### Statistical analysis and graph generation

The Perseus computational platform (v.1.6.7.0) (31) was used to perform Principal Component Analysis (PCA) and differential analysis on aggregated abundance tables. According to earlier reports (24), taxon, function, and taxon-specific function abundance data were subjected to binary logarithmic transformation to approximate a normal distribution (subsequently checked using the Kolmogorov-Smirnov test); features not reaching 70% of valid values in at least one group (for each comparison) were filtered out; missing values were replaced with a constant value, calculated as the binary logarithm of the lowest abundance value (approximated to the nearest integer) minus 1; differential abundance between groups was tested with a two-tailed paired Student’s *t*-test; the obtained *P*-values were corrected for multiple testing by calculating an FDR according to Benjamini and Hochberg (32), considering q = 0.05 as the significance threshold. Features mapped to fewer than three peptide sequences were filtered out from the final list of significant features.

A Kruskal-Wallis test with Dunn’s correction for multiple testing, available in GraphPad Prism (v.9.0.0), was performed to compare abundance ranking distributions.

Pearson correlations were calculated using an R script based on the *cor.test* function. Then, FDR was calculated using Benjamini-Hochberg’s correction for multiple testing.

Violin, scatter, and correlation plots were created using GraphPad Prism.

The lists of UniProt accession numbers corresponding to the significantly differential human proteins were imported in STRING (https://string-db.org) (33) and Reactome (https://reactome.org/) (34) to perform network and enrichment analyses, respectively. In STRING, a high confidence score (0.7), a high FDR stringency (1%), and MCL clustering (inflation = 3) were selected.

## RESULTS

### Experimental design and general metrics

Fecal samples were collected from 45 patients before (T0) and after (T1) BMS. Patients undergoing the two different surgical procedures were analyzed as a single group because their metabolic outcomes were comparable (17), and the sample size within each individual subgroup was insufficient to achieve adequate statistical power (35). After protein extraction and digestion, the obtained tryptic peptides were analyzed by LC-MS/MS. Database searching against a collection of human gut metagenomes and the *Homo sapiens* reference proteome yielded 101,602 non-redundant peptide sequences, comprising 94,691 microbial, 6,771 human, and 140 ambiguous peptides. Preliminary observations showed a significant increase at T1 in the microbial-to-human peptide abundance ratio (*P* = 0.033), suggesting a possible expansion of microbial biomass and/or reduction of the host components within the fecal samples. In subsequent analyses, microbial and human data sets were treated independently and subjected to distinct normalization procedures.

Microbial peptides were further subjected to taxonomic and functional annotation. Specifically, the percentages of microbial peptides that could be unambiguously annotated at the phylum, class, order, family, genus, and species levels of taxonomy were 69%, 66%, 65%, 47%, 45%, and 26%, respectively. Human peptides were grouped into 829 protein groups, each represented by a “master” protein.

**Fig 1:**
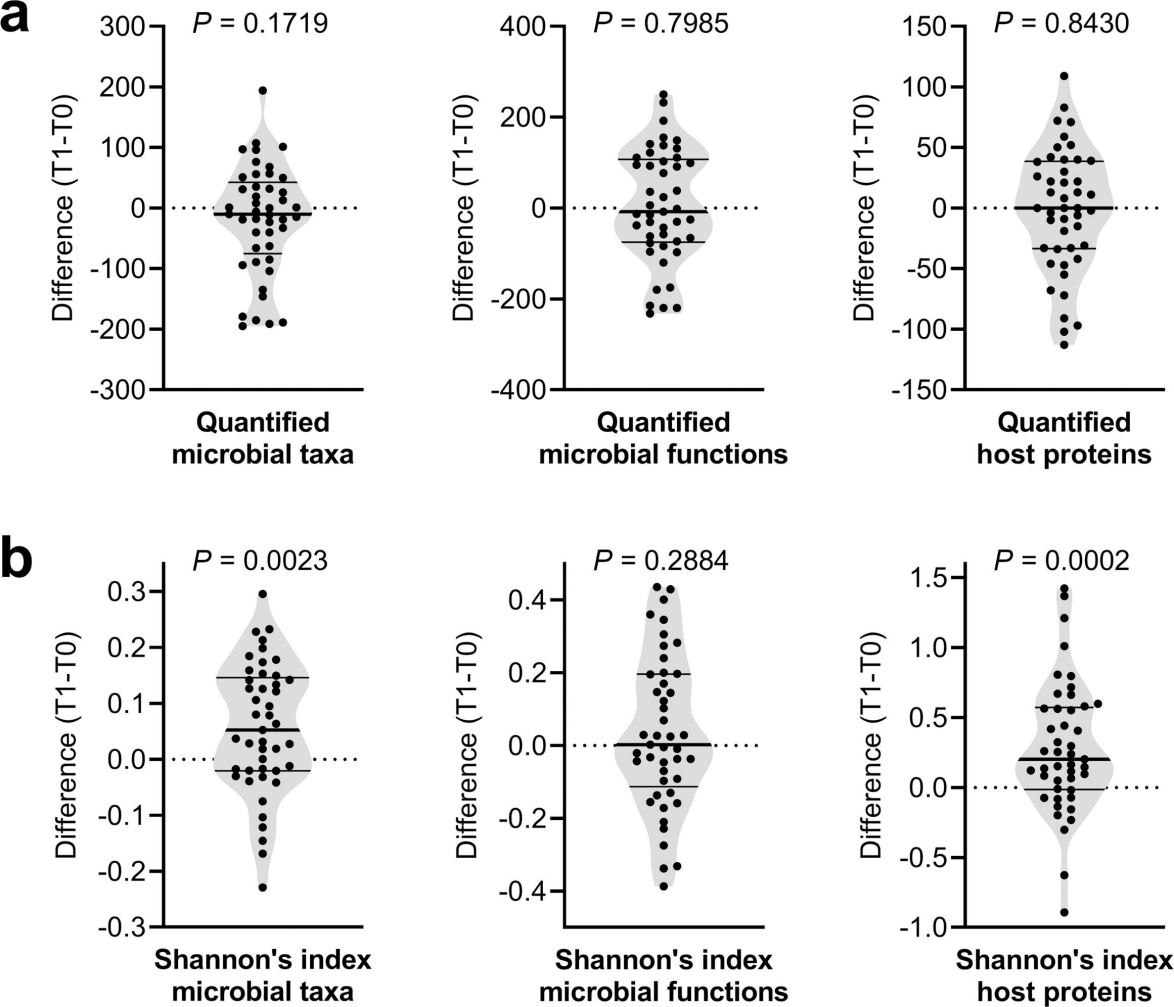
Richness (**a**) and alpha-diversity (**b**) measured based on microbial taxa (left), microbial functions (middle), and host proteins (right). Richness was calculated based on the number of features quantified in each sample, while alpha-at diversity was calculated according to Shannon’s index using the feature abundance distributions as inputs. Each dot indicates the difference between the values measured at T1 and T0 for a single patient. The horizontal thick black lines indicate the median of the distributions, while the thinner lines indicate the upper and lower quartiles. A dotted horizontal line indicates a difference in abundance between T1 and T0 equal to zero. The *P*-values obtained using the paired sample *t*-test are shown at the top of each violin plot.

Richness, alpha, and beta diversity were explored as preliminary metrics of the study to compare the general structure of the patients’ fecal metaproteomes at T1 compared to those at T0. As shown in Fig. 1a, no significant differences were observed between T1 and T0 in terms of the number of microbial taxa, microbial functions, or host proteins quantified. On the contrary, a significant increase in alpha-diversity was measured at T1, considering the abundance distribution of both microbial taxa and host proteins (Fig. 1b).

Beta diversity was also investigated via PCA. As shown in Fig. S1 to S3, no clear clustering could be found in PCA plots, possibly due to the well-known high interindividual variability in microbiome composition.

### Taxonomic changes in the fecal metaproteome after BMS

**Fig 2:**
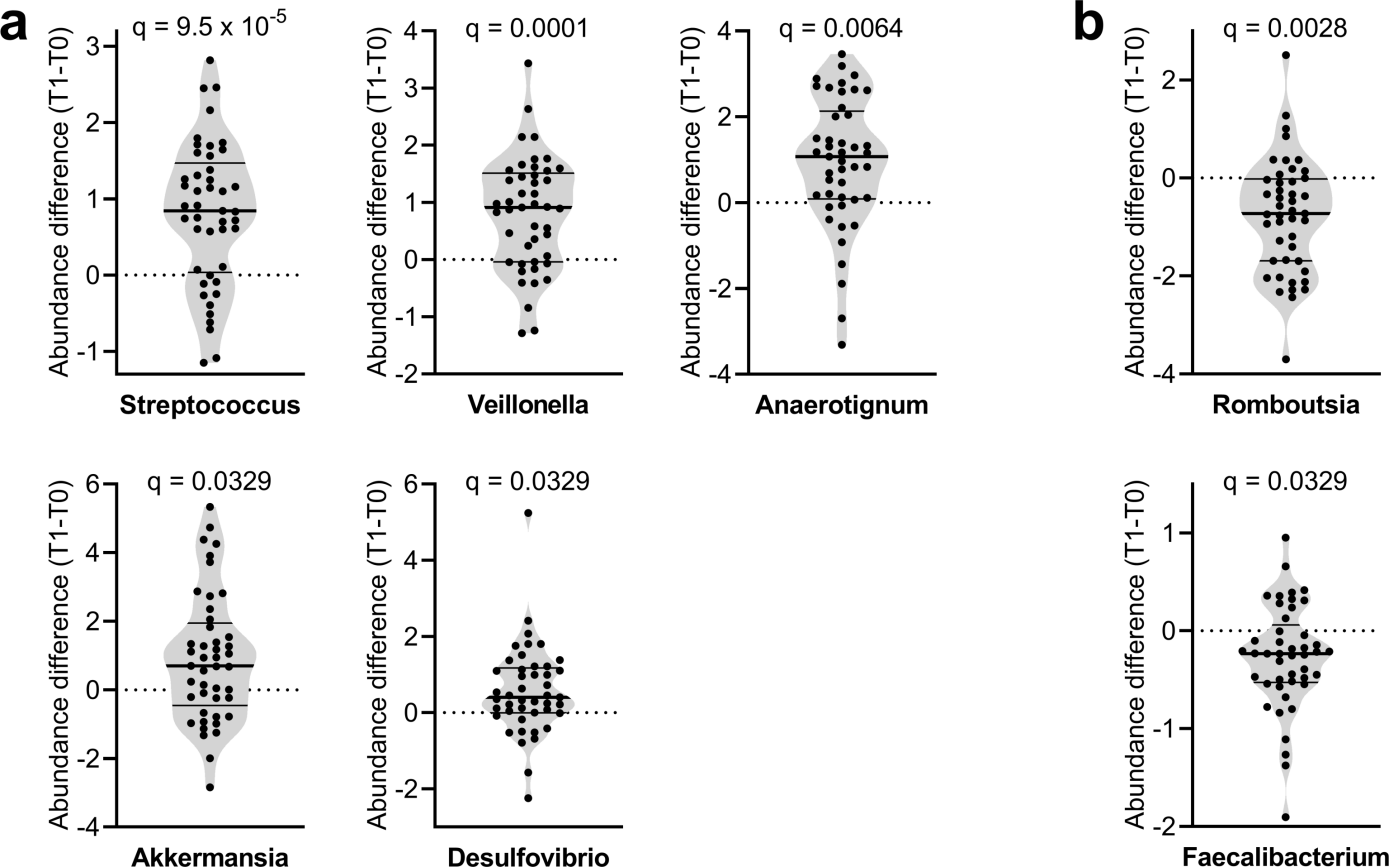
Bacterial genera with significantly different abundance between T1 and T0 in the analyzed patient cohort. Genera with higher abundance at T1 are shown in panel **a**, while those with higher abundance at T0 are shown in panel **b**. Each dot indicates the difference between the relative abundance values measured at T1 and T0 for a single patient. The horizontal thick black lines indicate the median of the distributions, while the thinner lines indicate the upper and lower quartiles. A dotted horizontal line indicates a difference in abundance between T1 and T0 equal to zero. The *q*-values obtained using the paired sample *t*-test and adjusted for multiple testing according to Benjamini and Hochberg are shown at the top of each violin plot.

Abundance values of microbial peptides were aggregated based on their taxonomic annotations. Accordingly, a total of 50 phyla, 62 classes, 112 orders, 157 families, 299 genera, and 469 species were detected. Then, taxonomic data were subjected to statistical analysis to determine which GM taxa had significantly changed their relative abundance after BMS. As a result, a significantly higher relative abundance at T1 was measured for 15 taxa, while five taxa were found to be significantly more abundant at T0. Focusing on the taxonomic rank of genus, which provides the optimal balance between taxonomic definition and annotation yield, *Akkermansia*, *Anaerotignum*, *Desulfovibrio*, *Streptococcus*, and *Veillonella* were found to be significantly more abundant at T1 compared to T0, while *Faecalibacterium* and *Romboutsia* showed the opposite behavior (Fig. 2). The list of all the taxa (at the various hierarchical levels) showing statistically significant differences in abundance between T1 and T0 is presented in Table S2.

### Functional changes in the fecal metaproteome after BMS

The abundance values of microbial peptides were also aggregated based on their KEGG KO annotations. A total of 1,801 non-redundant KO functions (regardless of taxonomic annotation) were detected. Overall, the number of phylum-, class-, order-, family-, and genus-specific KO functions was 2,427, 2,898, 3,122, 3,647, and 3,883, respectively. Functional data were subsequently subjected to statistical analysis to identify KO functions whose relative abundance significantly changed after BMS. All KO functions, across the different levels of taxonomic specificity, that showed statistically significant differences between T1 and T0 are listed in Table S3. To summarize the results and make them easier to illustrate graphically, KO functions that shared most or all peptides were grouped together. In each group, if the same KO function was significant at different levels of taxonomic specificity (e.g., phylum *Bacteroidota*, class *Bacteroidia*, and order *Bacteroidales*), the lowest level was selected (*Bacteroidales* in this example). After grouping, 28 KO functions significantly more abundant at T1 than at T0 and 6 KO functions significantly less abundant at T1 than at T0 were observed.

**Fig 3:**
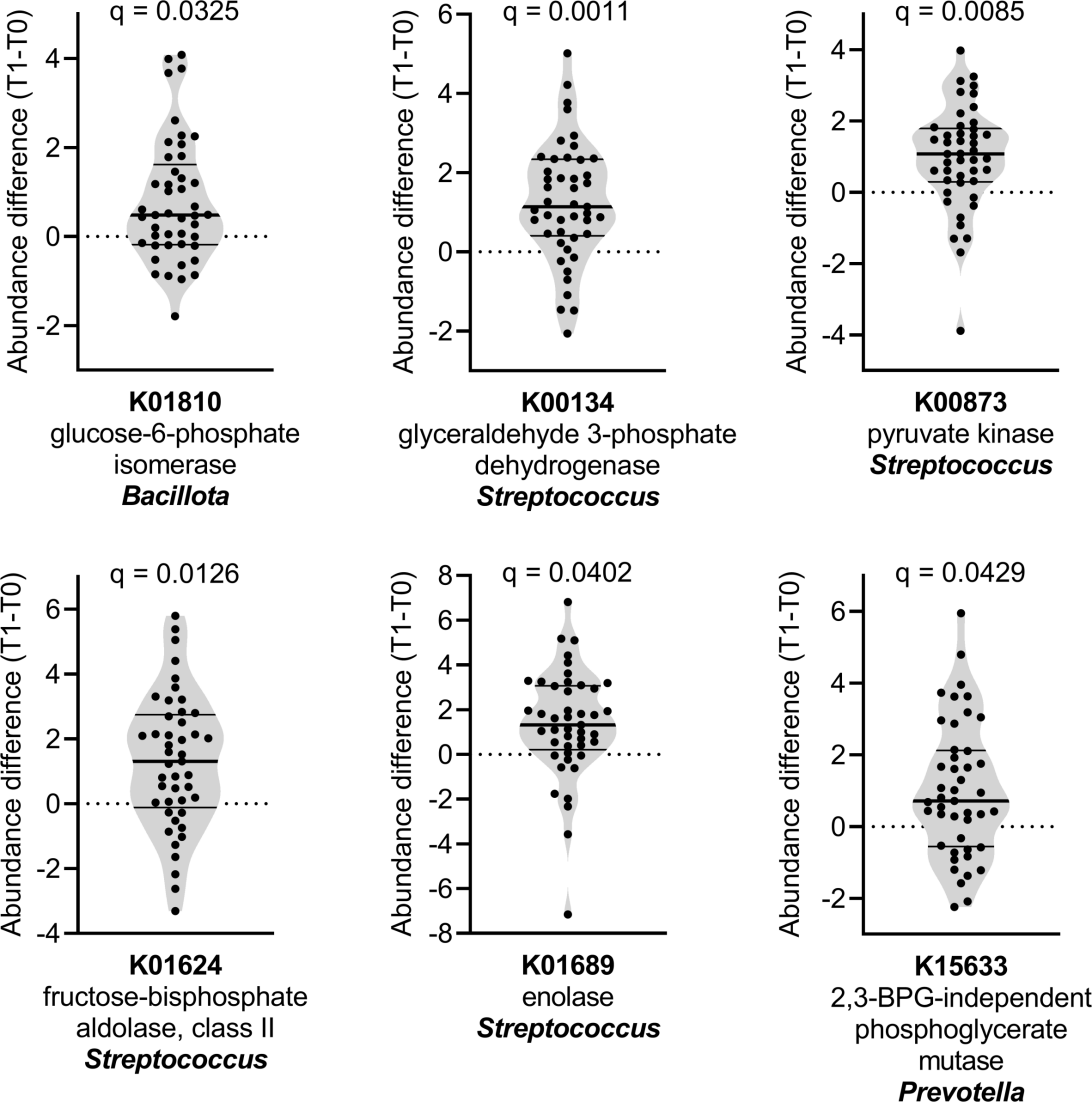
Taxon-specific KEGG KO functions belonging to the glycolysis pathway and showing a significantly higher abundance at T1 compared to T0 in the analyzed patient cohort. The lowest taxonomic annotation level associated with a significant difference was selected for each KO function. Each dot indicates the difference between the relative abundance values measured at T1 and T0 for a single patient. The horizontal thick black lines indicate the median of the distributions, while the thinner lines indicate the upper and lower quartiles. A dotted horizontal line indicates a difference in abundance between T1 and T0 equal to zero. The *q*-values obtained using the paired sample *t*-test and adjusted for multiple testing according to Benjamini and Hochberg are shown at the top of each violin plot. Functions are ordered by their KO number.

The 28 functions that were relatively higher at T1 were further grouped based on metabolic and biological categories according to the KEGG pathway classification system (with minor modifications). The first metabolic category included six glycolytic enzymes, as shown in Fig. 3. Four of them were unambiguously assigned to the bacterial genus *Streptococcus*, in line with taxonomic results. A phosphoglycerate mutase specifically expressed by members of the genus *Prevotella* was also significantly increased at T1. Finally, a glucose-6-phosphate isomerase encoded by members of the *Bacillota* phylum (mainly belonging to *Streptococcaceae* and *Oscillospiraceae*, although the abundance distribution of the peptide sequences assigned to these two families narrowly missed the significance threshold) was also included among the differential glycolytic enzymes.

**Fig 4:**
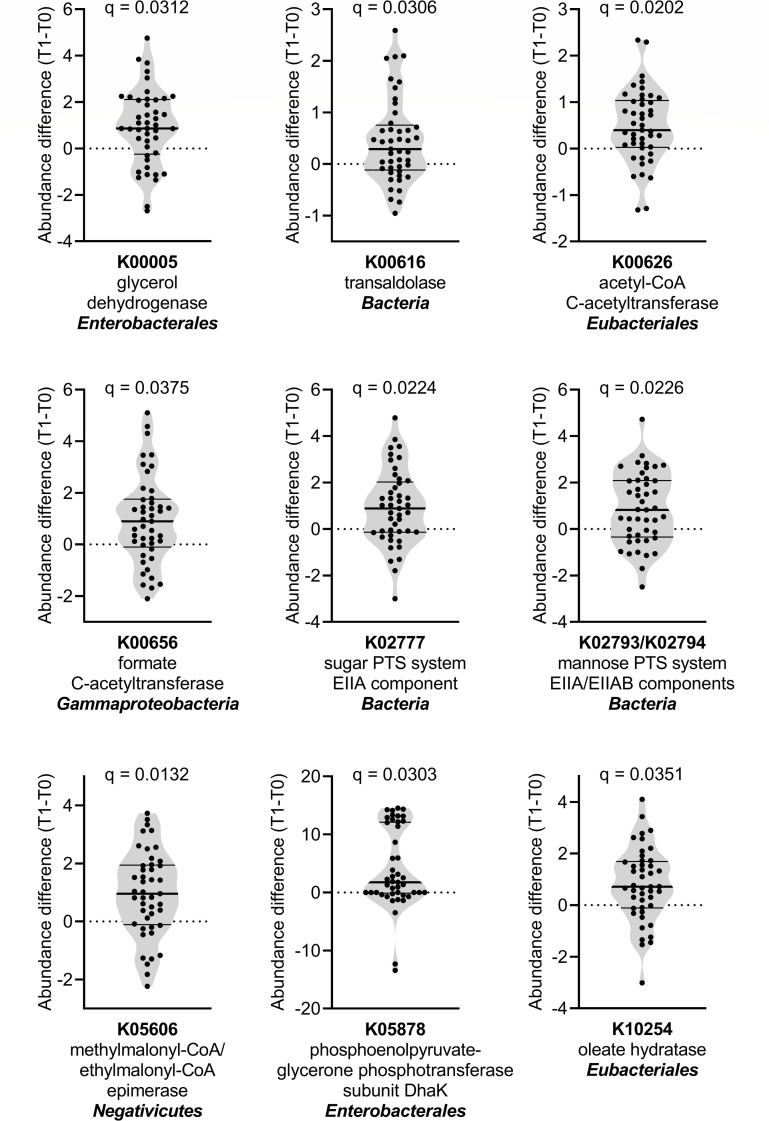
Taxon-specific KEGG KO functions belonging to carbohydrate metabolism pathways other than glycolysis and showing a significantly higher abundance at T1 compared to T0 in the analyzed patient cohort. The lowest taxonomic annotation level associated with a significant difference was selected for each KO function. Each dot indicates the difference between the relative abundance values measured at T1 and T0 for a single patient. The horizontal thick black lines indicate the median of the distributions, while the thinner lines indicate the upper and lower quartiles. A dotted horizontal line indicates a difference in abundance between T1 and T0 equal to zero. The *q*-values obtained using the paired sample *t*-test and adjusted for multiple testing according to Benjamini and Hochberg are shown at the top of each violin plot. Functions are ordered by their KO number.

The second category included nine protein functions involved in carbohydrate metabolism pathways other than glycolysis and showing a significantly higher relative abundance at T1, as illustrated in Fig. 4. Two of them were enzymes linking glucose and pyruvate metabolism with short-chain fatty acid biosynthesis, namely acetyl-CoA C-acetyltransferase and formate C-acetyltransferase. Specifically, the former was expressed by members of the order *Eubacteriales*, while the latter by members of the class *Gammaproteobacteria*. Furthermore, a typical propionogenic enzyme, methylmalonyl-CoA epimerase, encoded by members of the *Negativicutes* class, was significantly higher at T1. Enzymes related to lipid metabolism were also found, including two expressed by *Enterobacterales* and involved in glycerolipid metabolism (namely, glycerol dehydrogenase and PEP-glycerone phosphotransferase). The list also included two genetically conserved components of the phosphotransferase system, involved in sugar translocation into the bacterial cell, and transaldolase, a key enzyme of the pentose phosphate pathway.

**Fig 5:**
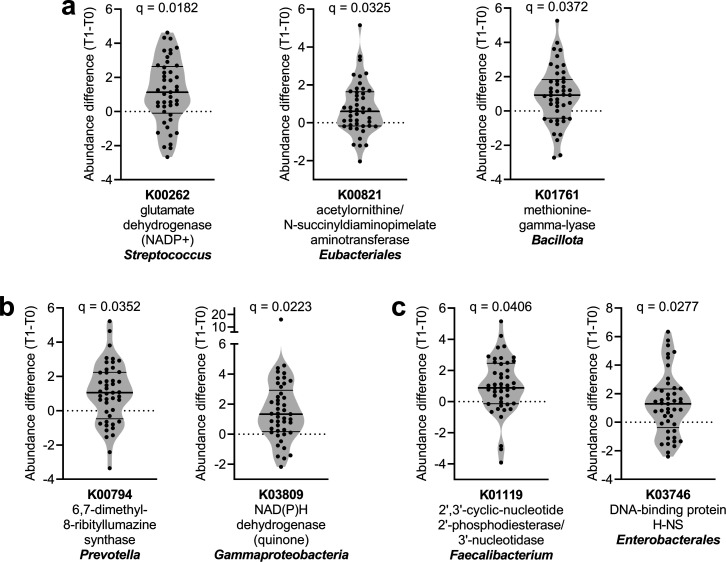
Taxon-specific KEGG KO functions belonging to amino acid metabolism (**a**), cofactor and vitamin metabolism (**b**), and nucleotide binding and metabolism (**c**) pathways and showing a significantly higher abundance at T1 compared to T0 in the analyzed patient cohort. The lowest taxonomic annotation level associated with a significant difference was selected for each KO function. Each dot indicates the difference between the relative abundance values measured at T1 and T0 for a single patient. The horizontal thick black lines indicate the median of the distributions, while the thinner lines indicate the upper and lower quartiles. A dotted horizontal line indicates a difference in abundance between T1 and T0 equal to zero. The *q*-values obtained using the paired sample *t*-test and adjusted for multiple testing according to Benjamini and Hochberg are shown at the top of each violin plot. Functions are ordered by their KO number.

Enzymes involved in amino acid metabolism that were found to be significantly higher in abundance at T1 are shown in Fig. 5a. These included the housekeeping glutamate dehydrogenase from *Streptococcus*, acetylornithine/N-succinyldiaminopimelate aminotransferase encoded by members of *Eubacteriales*, active in both the arginine and lysine biosynthetic pathways, and methionine-gamma-lyase expressed by *Bacillota*, relevant for methionine biosynthesis. Interestingly, most of the peptides of the latter two enzymes were assigned to members of the *Oscillospiraceae*/*Oscillibacter* lineage.

Two enzymes related to cofactor and vitamin metabolism were also included among the differential protein functions higher at T1, as depicted in Fig. 5b. Among them, 6,7-dimethyl-8-ribityllumazine synthase, expressed by *Prevotella*, is involved in riboflavin (vitamin B2) metabolism, whereas NAD(P)H-quinone dehydrogenase, encoded by members of *Gammaproteobacteria*, belongs to the ubiquinone (coenzyme Q10) biosynthesis pathway.

Among protein functions related to nucleotide binding and metabolism showing significantly higher abundance at T1 (Fig. 5c), we found a nucleotide phosphodiesterase/nucleotidase from *Faecalibacterium* and a DNA-binding protein from *Enterobacterales*.

**Fig 6:**
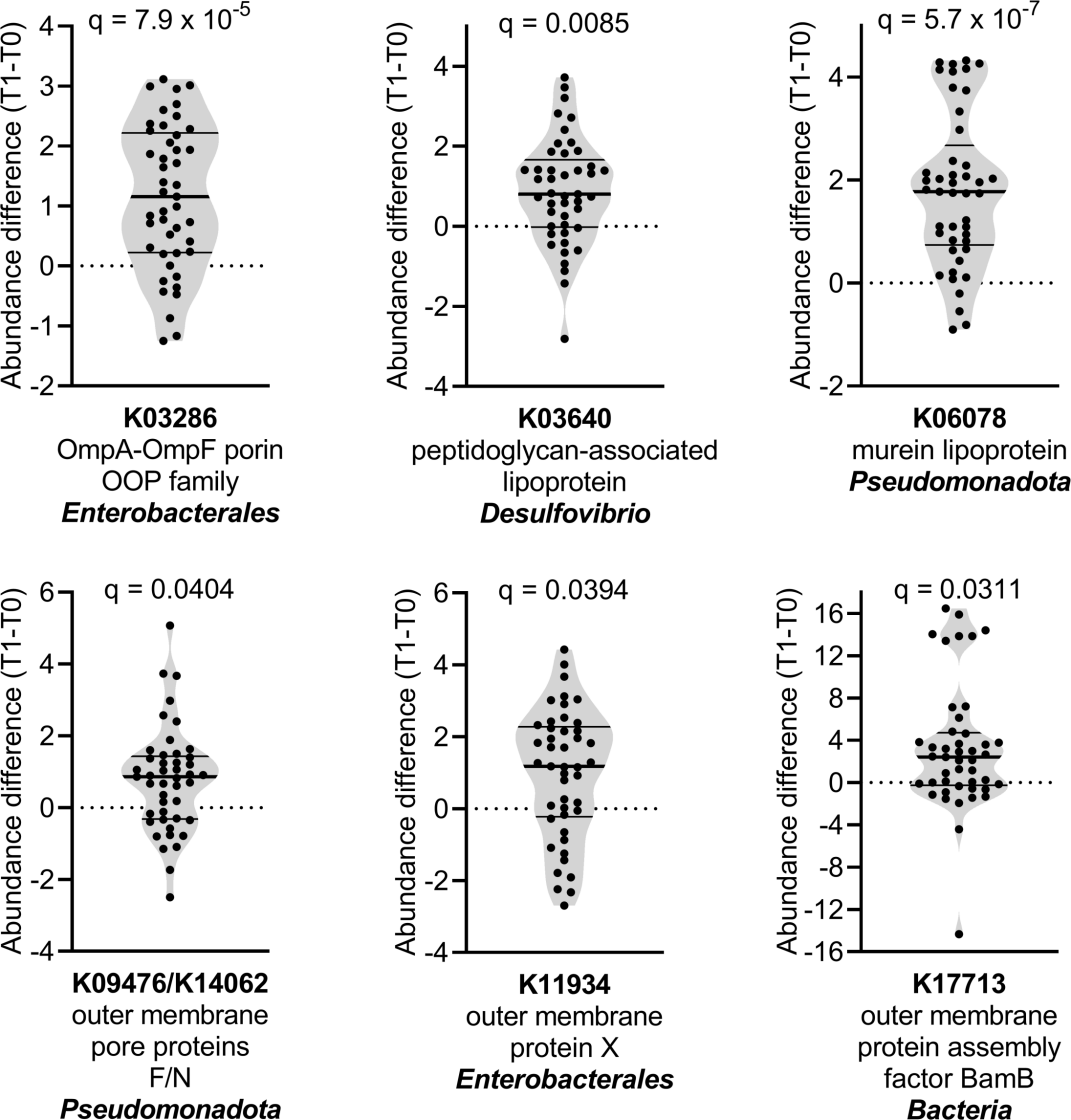
Taxon-specific KEGG KO functions belonging to the bacterial cell wall or membranes and showing a significantly higher abundance at T1 compared to T0 in the analyzed patient cohort. The lowest taxonomic annotation level associated with a significant difference was selected for each KO function. Each dot indicates the difference between the relative abundance values measured at T1 and T0 for a single patient. The horizontal thick black lines indicate the median of the distributions, while the thinner lines indicate the upper and lower quartiles. A dotted horizontal line indicates a difference in abundance between T1 and T0 equal to zero. The *q*-values obtained using the paired sample *t*-test and adjusted for multiple testing according to Benjamini and Hochberg are shown at the top of each violin plot. Functions are ordered by their KO number.

Finally, six of the differential KO functions higher in abundance at T1 belonged to the cell wall and the outer membrane of gram-negative bacteria, as shown in Fig. 6. These included murein lipoprotein and several porins, which could be assigned to the *Pseudomonadota*/*Gammaproteobacteria*/*Enterobacterales*/*Enterobacteriaceae* lineage (at different levels of depth and significance). The peptidoglycan-associated lipoprotein encoded by *Desulfovibrio* also exhibited a significant increase in relative abundance at T1 compared to T0.

**Fig 7:**
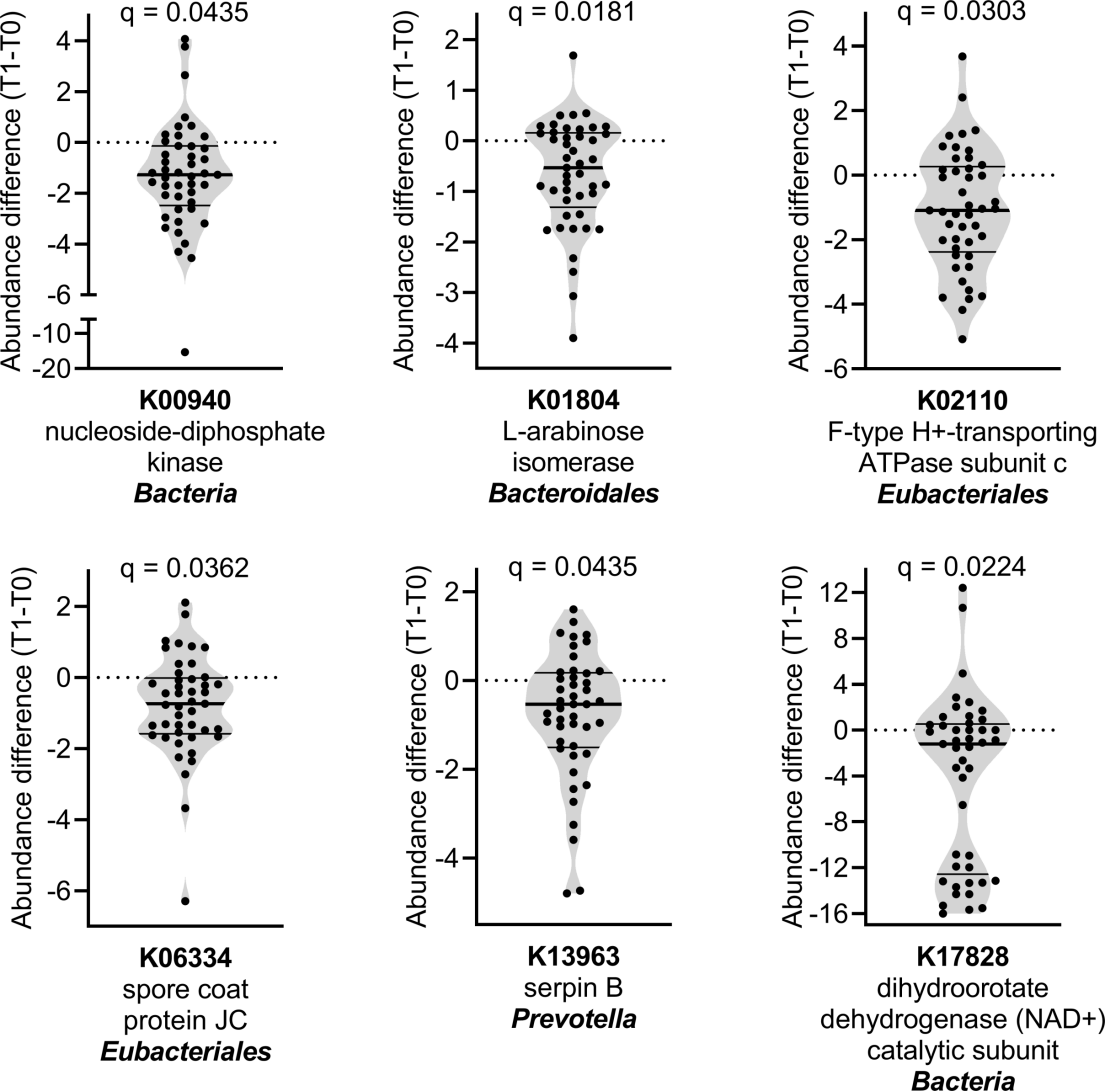
Taxon-specific KEGG KO showing a significantly higher abundance at T0 compared to T1 in the analyzed patient cohort. The lowest taxonomic annotation level associated with a significant difference was selected for each KO function. Each dot indicates the difference between the relative abundance values measured at T1 and T0 for a single patient. The horizontal thick black lines indicate the median of the distributions, while the thinner lines indicate the upper and lower quartiles. A dotted horizontal line indicates a difference in abundance between T1 and T0 equal to zero. The *q*-values obtained using the paired sample *t*-test and adjusted for multiple testing according to Benjamini and Hochberg are shown at the top of each violin plot. Functions are ordered by their KO number.

Among the protein functions with significantly higher abundance at T0 compared to T1 (Fig. 7), we found two well-conserved enzymes involved in nucleotide metabolism (namely, nucleoside-diphosphate kinase and dihydroorotate dehydrogenase), a subunit of the F-type ATPase, and a spore coat protein, both expressed by *Eubacteriales*, and two proteins encoded by members of *Bacteroidota*, specifically L-arabinose isomerase from *Bacteroidales* and a serine-protease inhibitor (serpin) from *Prevotella*.

### Comparison with fecal metaproteome features from healthy subjects

**Fig 8:**
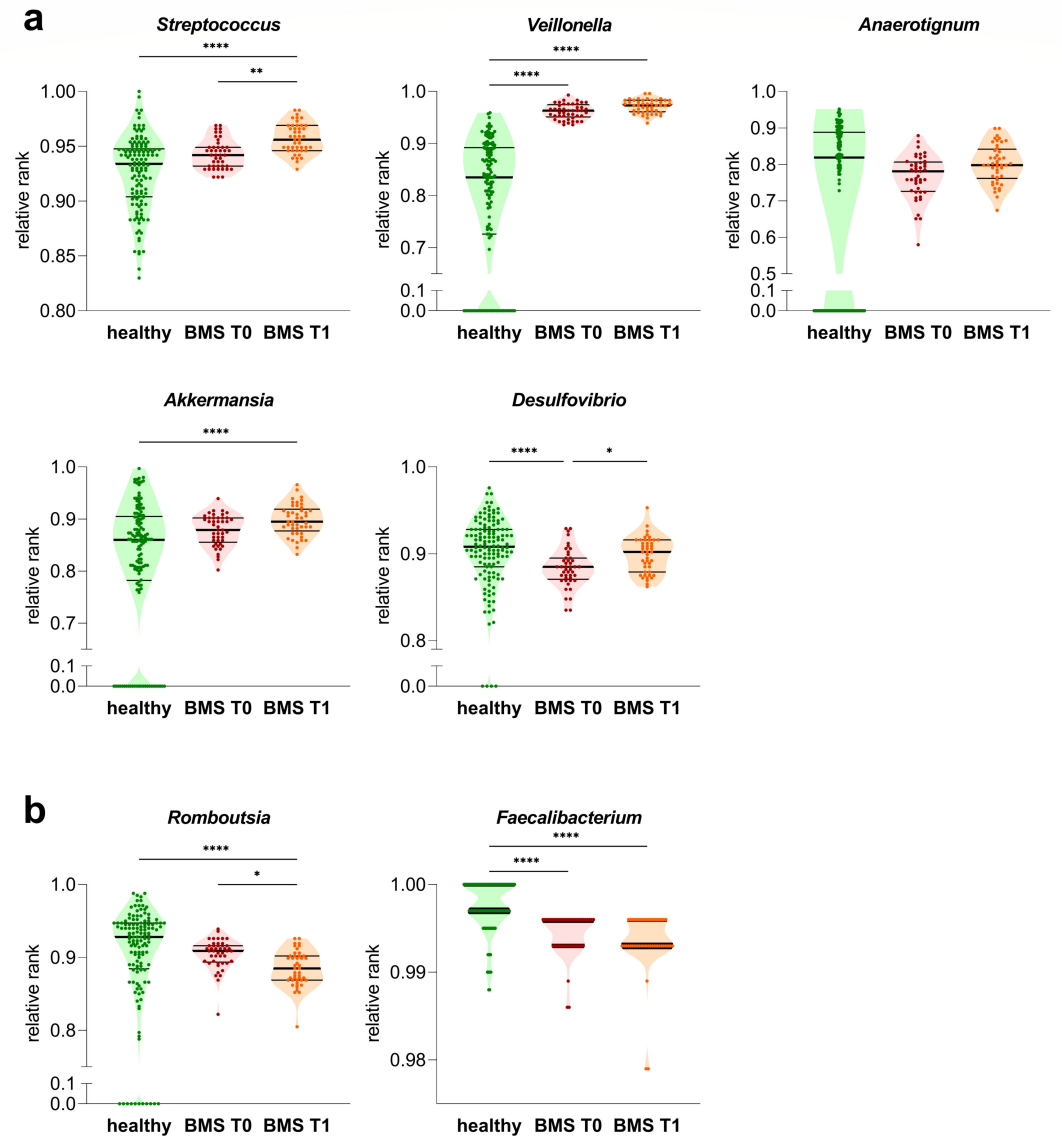
Relative abundance ranking distribution of microbial genera shown in Fig. 2, as measured in this study (BMS T0 and BMS T1) and in a collection of fecal metaproteome data sets from healthy individuals (healthy). Genera with higher abundance at T1 are shown in panel **a**, while those with higher abundance at T0 are shown in panel **b**. Each circle represents an individual subject/patient. The horizontal thick black lines indicate the median of the distributions, while the thinner lines indicate the upper and lower quartiles. Statistical significance was calculated using a Kruskal-Wallis test with Dunn’s correction for multiple comparisons (*P < 0.05; **P < 0.01; ****P < 0.0001).

Then, we aimed to contextualize the abundance variations in taxa and functions observed in BMS patients relative to the general population. To this purpose, we compared the relative ranking distributions of the microbial taxonomic and functional features measured in this study with those measured in a published meta-analysis of fecal metaproteomics data sets from 134 healthy individuals (36). Relative ranking was preferred over relative abundance measures to minimize potential batch effects arising from variability in sample preparation and instrumentation across data sets. Bacterial genera found to be significantly more abundant at T1 than at T0 in BMS patients in this study (Fig. 2a) exhibited different relative abundance ranking trends. As shown in Fig. 8a, the relative abundance ranks of *Streptococcus* and *Akkermansia* in BMS patients at T0 were comparable to those measured in healthy subjects, whereas those measured at T1 were significantly higher. Furthermore, the relative abundance rank of *Veillonella* in BMS patients was significantly higher than that measured in healthy subjects, both at T0 and T1. Conversely, the relative abundance rank of *Desulfovibrio* was significantly lower in BMS patients at T0 compared to healthy subjects but recovered substantially at T1. Finally, *Faecalibacterium* and *Romboutsia*, whose relative abundance decreased at T1 (Fig. 2b), showed lower relative abundance ranks in BMS patients compared to healthy subjects; this difference was significant at both time points for *Faecalibacterium,* but only at T1 for *Romboutsia* (Fig. 8b).

Regarding taxon-specific KEGG KO functions, we limited the comparison to those that changed significantly in abundance between T1 and T0 (Fig. 3 to 7) and for which abundance data were available in the “healthy” data set (i.e., generic, phylum-specific, and genus-specific KOs). Most KOs that significantly increased at T1 compared to T0 also showed significantly higher abundance ranks in BMS patients than in healthy subjects, particularly for functions encoded by *Streptococcus* (Fig. S4 and S7). Interestingly, in nearly all these cases, the T1 abundance rank was even more divergent from the healthy data set rank than the T0 rank. Conversely, glucose-6-phosphate isomerase, expressed by members of *Bacillota*, as well as transaldolase and the mannose PTS system generically assigned to bacteria, exhibited significantly lower abundance ranks in BMS patients than in healthy subjects, with ranks measured at T1 approaching those of the healthy cohort (Fig. S4 and S5). Regarding functions detected at significantly higher levels at T0 than at T1 in this study (Fig. S8), bacterial nucleoside-diphosphate kinase and dihydroorotate dehydrogenase exhibited significantly lower abundance ranks in BMS patients compared to healthy subjects, with the T1 rank deviating even further from the healthy cohort than T0. In contrast, serpin B encoded by *Prevotella* showed a completely opposite trend.

### Human fecal proteins changing in abundance after BMS

**Fig 9:**
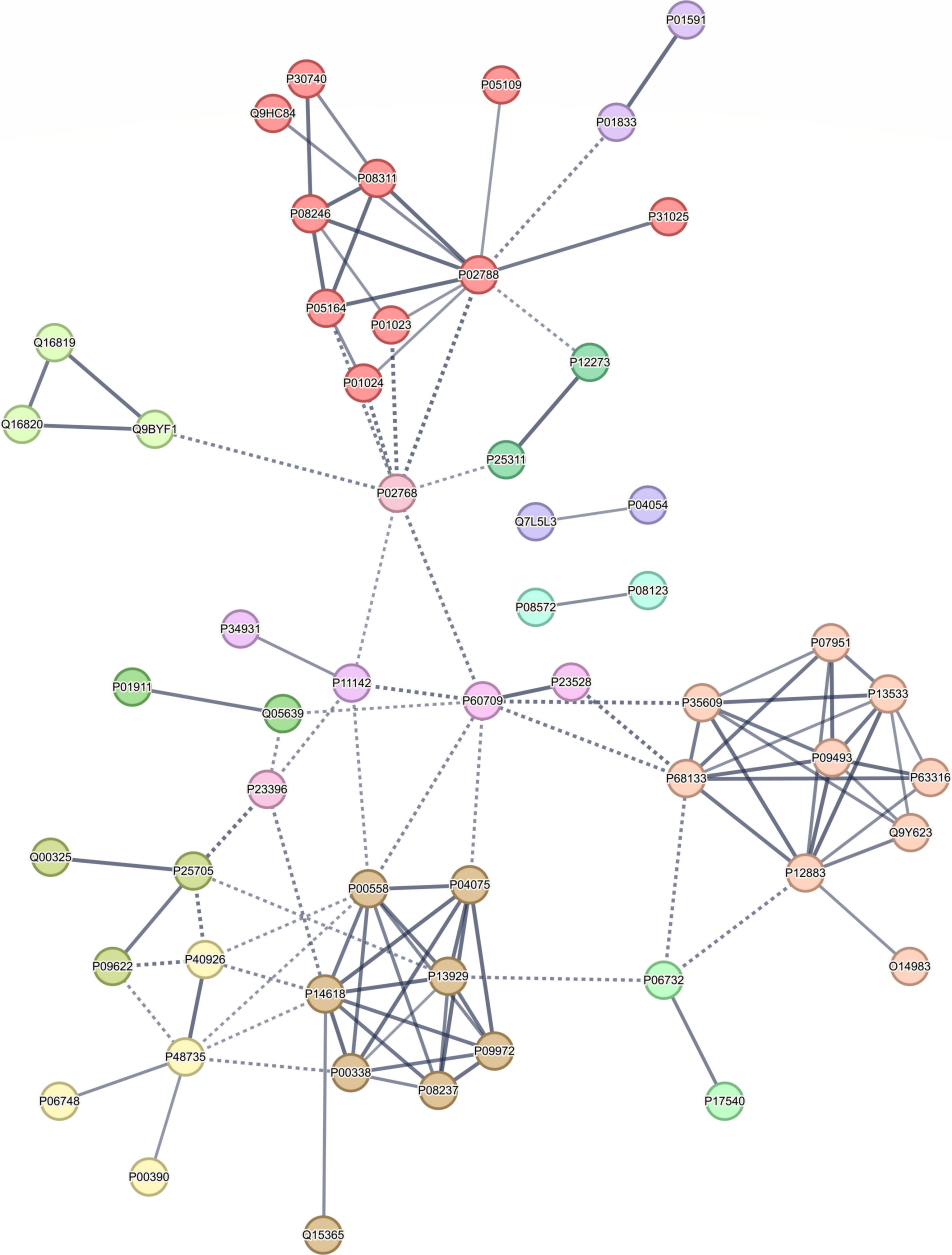
STRING networks based on host proteins with significantly higher abundance at T1 compared to T0. The main protein networks were related to defense response (red), muscle contraction (apricot), glycolysis (beige), and citrate cycle (lemon). See Table S4 for detailed information about the color code and protein accession numbers associated with each network.

Eighty-eight human proteins detected in the fecal samples were found to be differentially expressed between the two time points analyzed, based on the statistical analysis results. More specifically, 74 of them were significantly more abundant at T1 compared to T0, while the remaining 14 followed the opposite trend, as detailed in Table S4. Enrichment analysis of proteins higher in T1 revealed several significantly enriched functional pathways, including neutrophil degranulation, glycolysis, muscle contraction, and the immune system. Many of these proteins are known to interact and form molecular networks, as illustrated in Fig. 9.

On the other hand, most of the proteins higher in T0 were enzymes secreted by the pancreas and involved in protein digestion, along with other functions related to immunity. These proteins are listed in Table 1.

**TABLE 1:** Human proteins with significantly higher abundance in T0 compared to T1^*a*^

UniProt accession	Protein name	*q*-value	Difference (T0 − T1)
P13473	Lysosome-associated membrane glycoprotein 2	0.00,358	5.390
A0A0A0MT69	Immunoglobulin kappa joining 4 (fragment)	0.03,700	3.167
P15085	Carboxypeptidase A1	0.00,001	1.794
Q9BYE9	Cadherin-related family member 2	0.00,001	1.479
P15086	Carboxypeptidase B	0.00,225	1.159
P01009	Alpha-1-antitrypsin	0.00,256	1.123
P07477	Trypsin-1	0.00,225	1.033
P09093	Chymotrypsin-like elastase family member 3A	0.00,001	1.012
Q99895	Chymotrypsin-C	0.00,347	0.977
Q6UWV6	Ectonucleotide pyrophosphatase/phosphodiesterase family member 7	0.00,097	0.859
P0DP04	Immunoglobulin heavy variable 3-43D	0.03,203	0.826
P09923	Intestinal-type alkaline phosphatase	0.00,212	0.814
P01602	Immunoglobulin kappa variable 1-5	0.01,825	0.794
Q9UGM3	Deleted in malignant brain tumors 1 protein	0.01,825	0.609

^*a*^ Difference values and *q*-values were obtained by paired sample Student’s *t*-test followed by Benjamini and Hochberg’s adjustment for multiple testing. Proteins are ordered by decreasing difference value.

### Correlation between metaproteome features and clinical parameters

Finally, the main metaproteome features measured in this study (namely, abundances of microbial phyla and genera; generic, phylum-specific, and genus-specific KO functions; and host proteins) were correlated with patients' clinical parameters, including BMI and excess BMI loss values (Table S1), as well as several anthropometric and blood parameters (Table S5). More specifically, metaproteome features at T0 were correlated both with clinical parameters at T0 and with their relative variation between T1 and T0; notably, the latter correlations may have predictive value. Furthermore, metaproteome features at T1 were correlated with clinical parameters at the same time point, as well as with their variation between T1 and T0.

**Fig 10:**
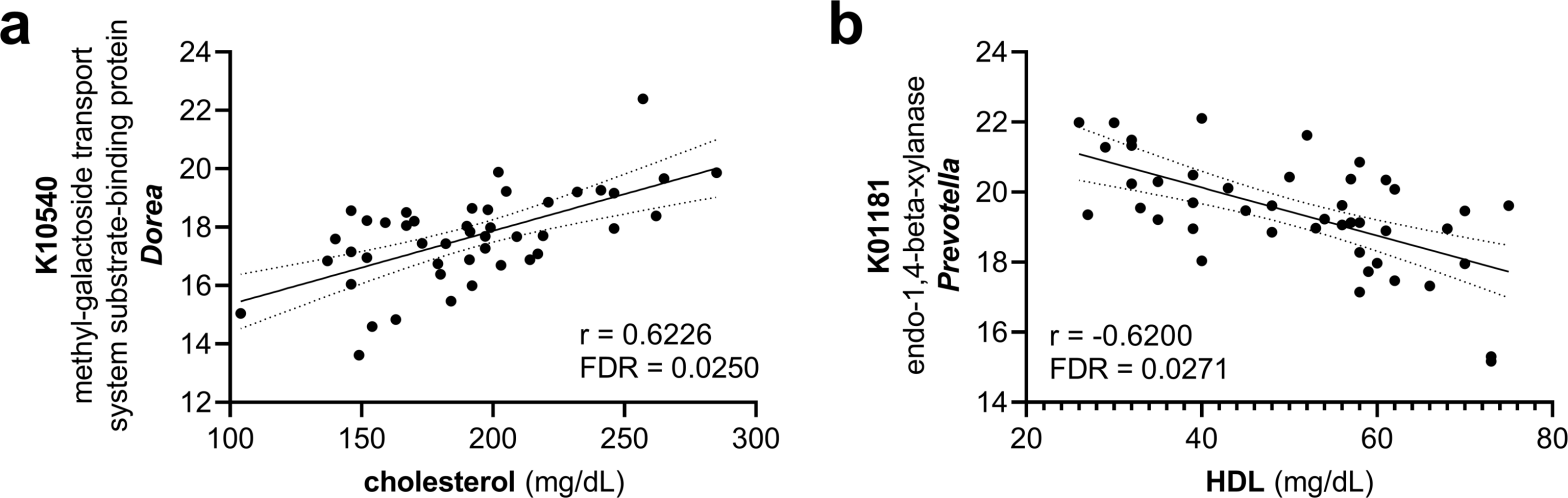
Scatter plots showing significant Pearson correlations between taxon-specific KEGG KO functions and patients’ clinical parameters. Linear regression lines (mean and 95% CI) are shown. The Pearson correlation coefficient (R) and the false discovery rate (FDR), calculated according to Benjamini-Hochberg’s correction for multiple testing, are also shown. (**a**) Correlation between the log-transformed relative abundance of methyl-galactoside transport system substrate-binding protein encoded by *Dorea*, as measured in patients’ fecal metaproteomes at T0, and cholesterol, as measured in patients’ blood at T0. (**b**) Correlation between the log-transformed relative abundance of endo-1,4-beta-xylanase encoded by *Prevotella*, as measured in patients' fecal metaproteomes at T1, and HDL, as measured in patients' blood at T1.

As shown in Fig. 10, the fecal amount of methyl-galactoside transport system substrate-binding protein encoded by *Dorea* measured at T0 showed a significant positive correlation with blood cholesterol concentration measured at T0, while the fecal amount of endo-1,4-beta-xylanase encoded by *Prevotella* measured at T1 showed a significant negative correlation with blood HDL concentration measured at T1. Moreover, a positive correlation was observed between the fecal amount of nitrogen fixation protein NifU (with generic taxonomic assignment to bacteria) measured at T1 and the relative variation of the blood cortisol concentration between T1 and T0 (Fig. S9). No significant correlations with potential predictive value were found between the metaproteome features measured at T0 and excess BMI loss (i.e., the primary BMS outcome).

## DISCUSSION

Metaproteomic analysis of a longitudinal sample set from 45 obese patients successfully enabled us to capture the dynamic functional changes that occur in the human GM after BMS.

To the best of our knowledge, only one prior metaproteomic study has explored changes in GM functions following BMS in human patients. In that study, Sanchez-Carrillo et al. examined a cohort of 40 patients at baseline and at 1 and 3 months post-RYGB or VSG, using a pooling approach in which two fecal sample pools were created from 20 individuals each. In contrast, a key strength of our study is the use of an individualized, longitudinal design that includes both baseline and 2-year post-surgery samples for all 45 participants. This approach improves the reliability and resolution of our findings compared to pooled sample studies and significantly expands the current understanding of long-term functional changes in the GM after BMS. Additionally, all patients in our study underwent comparable surgical procedures (RYGB or OAGB) that share similar anatomical and physiological characteristics, specifically the resection of the proximal jejunum. This uniformity resulted in consistent clinical outcomes, including comparable excess weight loss and improvements in key metabolic markers (17). Importantly, this study also presents matched proteomic data from both the GM and host, revealing significant functional shifts associated with biliopancreatic secretions 2 years post-surgery.

The first particularly intriguing finding from our analysis is that metaproteomic data robustly recapitulate previous observations regarding taxonomic changes obtained via DNA sequencing approaches. It is well established that metaproteomics, which combines taxonomic and functional information, can provide an accurate measure of the contribution of each taxon to the total microbial biomass within a particular sample (37). In a complex microbial community, certain taxa can maintain a constant biomass while showing changes in the abundance of specific protein functions. Conversely, others can vary their contribution to the total biomass over time, implying a change in the relative abundance of their whole proteome within the global community metaproteome. Investigating the metaproteome profile with a focus on taxon-specific functions, as in this study, may help determine whether a certain biological function correlates with the global abundance trend of the taxon responsible for it or follows an independent trajectory (36, 38, 39). Furthermore, several published studies have demonstrated that the functional profile of the fecal metaproteome remains relatively stable over time (40–42). This supports the reliability of comparing pre- and post-surgery fecal profiles within the same patient via a longitudinal design, as employed in this work.

Although previous metagenomic studies reported somewhat different taxonomic outcomes—likely explained by the lack of fully standardized methodologies for sample collection and storage, DNA extraction and sequencing, and bioinformatic analysis—a common variation observed after RYGB and/or OAGB is an increase in GM alpha-diversity (43–45). The two components of alpha-diversity, richness and evenness, reflect the loss or gain of taxa and ecological balance, respectively. Our metaproteomic analysis showed an increase in alpha-diversity after BMS, despite a stable richness of microbial taxa. This indicates a reorganization of microbial abundances rather than the addition or loss of taxa, reflecting greater ecosystem stability, functional redundancy, and potentially improved host-microbiome interactions post-BMS. Higher GM alpha-diversity values may result from modified gut architecture and digestive ecology, as well as adherence to the recommended post-BMS healthy diet (46).

Other consistent variations observed after BMS in previous DNA sequencing-based studies include the relative increase of the bacterial genera *Akkermansia*, *Veillonella*, and *Streptococcus*, together with members of the *Pseudomonadota* phylum (14, 45, 47, 48). Strikingly, the keystone GM genus *Faecalibacterium* is consistently among the very few genera reported to decrease after surgery (7, 11, 49). Here, we confirmed by metaproteomics that the relative abundances of *Akkermansia*, *Veillonella*, and *Streptococcus* are significantly increased up to 2 years after BMS. Increased *Akkermansia* can be explained by reduced caloric intake and dietary shifts following BMS, since this genus utilizes host-derived substrates rather than dietary ones (23, 50). However, microbial alterations observed following RYGB and OAGB cannot be ascribed solely to reductions in caloric intake or nutrient absorption (8). The rapid delivery of meals into the jejunum and the shortening of the small intestine lead to profound physicochemical environmental changes that, in turn, are expected to alter the spatial distribution of microbial taxa, with a relative enrichment of fermentative and aerotolerant species in distal segments (8, 51). This can explain the significant increase in the relative abundance of *Streptococcus* within the metaproteome after BMS, which our analysis links mainly to glycolytic activity. Furthermore, this finding is consistent with the significantly higher relative abundance of its syntrophic genus *Veillonella*, whose growth is usually supported by the lactate produced by *Streptococcus* (52). The main fermentation products of *Veillonella*, in turn, are acetate and propionate (53). Consistent with this, our data show a significantly increased abundance at T1 of methylmalonyl-CoA/ethylmalonyl-CoA epimerase encoded by *Negativicutes* (the class to which *Veillonella* belongs). This enzyme is key to the succinate pathway, one of the main bacterial routes for propionate production. We also observed a concurrent, significant increase in the relative abundance of *Anaerotignus* (a member of the *Lachnospiraceae* family) at T1. This genus also grows on lactate and produces predominantly acetate and propionate as fermentation end-products (54); notably, the most abundant protein detected for this bacterial genus in our study was propionate CoA-transferase. This functional evidence is of particular interest 2 years post-BMS, given the multifaceted roles of propionate: it influences metabolic regulation by affecting hepatic gluconeogenesis and lipogenesis, modulates appetites via the release of anorexigenic hormones (PYY and GLP-1), and, more broadly, regulates transcription with significant implications for host physiopathology (55–57).

*Desulfovibrio* was also significantly increased in our cohort of BMS patients at T1. It is well established that the synthesis and circulation of bile acids (BAs), as well as their signaling pathways, undergo substantial changes after BMS. These include the rapid delivery of undiluted bile to the distal ileum and the colon, which partially contrasts with the increased pool size of reabsorbed BAs and BA-activated secretion of satiety gut hormones GLP-1 and PYY (58, 59). In mice, *Desulfovibrio* has been linked to enhanced cecal production of secondary BAs and increased BA hydrophobicity, which, in turn, facilitates intestinal cholesterol absorption (60). Moreover, *Desulfovibrio*-derived H_2_S has been shown to activate hepatic FXR and suppress CYP7A1 expression (60), affecting BA metabolism and promoting gallstone development (60). Notably, gallstones are a common complication of BMS (61). Taken together, these observations suggest that *Desulfovibrio* abundance could represent a potential target for reducing gallstone risk following BMS.

Variations in the abundance of *Faecalibacterium* are of particular interest, as this genus is a keystone member of the GM, consistently detected in healthy human metaproteomes and valued as the top contributor to butyrate biosynthesis (36). Cross-sectional studies investigating its relative abundance in lean and obese individuals have yielded conflicting data. While most studies do not report significant changes, some showed a lower abundance of *Faecalibacterium* in obese compared to lean adults (44, 62, 63); conversely, others reported increased abundance in obese children and adults (64–69) or a reduced butyrate-producing capacity in lean subjects (70). Interestingly, in longitudinal studies, decreased abundance of *Faecalibacterium* correlates with weight loss as early as 3 months after BMS (7, 11, 49). The increased concentration of BAs, including chenodeoxycholic acid (CDCA) and deoxycholic acid (DCA), well documented in humans and animal models, can contribute to the lowered abundance of *Faecalibacterium* (71). *Faecalibacterium*, unlike *Akkermansia* or members of *Pseudomonadota*, is inhibited by CDCA and DCA (72). Furthermore, DCA can promote the growth of *Akkermansia muciniphila in vivo* (72). Our metaproteomic approach confirmed a significantly lower proportion of *Faecalibacterium* in the fecal metaproteome 2 years after BMS. Other changes in post-intervention nutrition can induce physiological changes to which *Faecalibacterium* has adapted (7, 49). Strikingly, we identified 2′,3′-cyclic-nucleotide 2′-phosphodiesterase/3′-nucleotidase as the sole function encoded by *Faecalibacterium* found to be significantly increased in relative abundance 2 years post-BMS. This suggests that *Faecalibacterium* may use this enzyme to reclaim nucleosides as building blocks or energy sources (nucleotide salvage pathway) as a form of adaptation to a low-nutrient and more competitive gut environment.

Together with *Faecalibacterium*, only *Romboutsia*, a spore-forming bacterium belonging to *Peptostreptococcales*, showed significantly lower abundance at T1. Interestingly, *Intestinibacter*, also belonging to *Peptostreptococcales*, was less abundant at T1 with significance approaching the threshold (q-value 0.056). These results are consistent with our previous metaproteomic study on GM modification after obesogenic *ad libitum* feeding in rats (23) and with a metagenomics-based human investigation demonstrating a profound reduction of *Romboutsia* after RYGB, linked with a decrease in glycerophospholipids implicated in obesity-induced fatty liver disease (12).

In this study, we aimed to contextualize the changes in gut microbiota composition and function observed in BMS patients within the broader framework of the general population. To this end, we compared our data with a recent meta-analysis of fecal metaproteome data sets from healthy subjects across diverse global cohorts (36). Although this collection of data sets cannot be considered a true control group, given that the metaproteomic profiles were generated using heterogeneous experimental and analytical methods that are not fully comparable with those used in the present study, the main result of the comparison is nonetheless noteworthy. Specifically, in most cases, the abundance ranks measured at T1 in BMS patients deviated further from the healthy population than those measured at T0. This suggests that post-operative microbiome remodeling, driven by anatomical, physiological, metabolic, and nutritional factors, leads to a structural and functional equilibrium that diverges from that of a healthy, normal-weight population. Further studies involving matched control groups are needed to validate these observations and better elucidate their biological significance. Finally, it should be noted that the statistical test used to compare ranking data with the healthy population (Kruskal-Wallis test) does not account for the paired nature of pre- and post-BMS data. Consequently, some comparisons between T0 and T1 appeared non-significant in this context, despite having reached significance in the previous paired-data analysis. Therefore, the results of the paired analysis should be considered the primary and most reliable reference for the comparison between T0 and T1 in BMS patients.

Two bacterial enzymes involved in specific nutrient-absorption pathways correlated significantly with patients’ clinical data: the methyl-galactoside transport system substrate-binding protein encoded by *Dorea* correlated positively with cholesterol at T0, while endo-1,4-beta-xylanase encoded by *Prevotella* correlated negatively with HDL at T1. Specifically, changes in the abundance of the methyl-galactoside transport system substrate-binding protein are likely to be induced by either greater galactose availability or reduced availability of alternative sugars. Conversely, variations in endo-1,4-beta-xylanase abundance may reflect changes in the intake of xylan-rich dietary fibers. Although our current data cannot establish a direct mechanistic link between these microbial pathways and host cholesterol metabolism, the robustness of these associations underscores the potential of metaproteomics to identify microbial biomarkers associated with cholesterol profiles in BMS patients.

Animal models offer valuable insights into metaproteomic changes following BMS, holding strong translational potential for human medicine. In a pioneering study, Haange et al. reported increased levels of acetylornithine deacetylase, ornithine carbamoyltransferase, and glutamate dehydrogenase in the GM of post-BMS mice (73). Similarly, in our human cohort, we observed a marked increase in glutamate dehydrogenase, along with elevated levels of acetylornithine/N-succinyldiaminopimelate aminotransferase, an enzyme also involved in arginine biosynthesis.

Although no taxa within the *Bacteroidota* phylum exhibited significant changes in abundance at T1, the relative abundances of specific proteins encoded by members of this phylum decreased significantly at T1, including L-arabinose isomerase assigned to *Bacteroidales* and serpin B assigned to *Prevotella*. Post-surgery, L-arabinose isomerase may be downregulated due to a lower intake of arabinose-rich fibers. Notably, serpin B is a serine-protease inhibitor that *Prevotella* may produce as a defense mechanism against cell damage and inflammation caused by host digestive enzymes, such as trypsin and chymotrypsin-like elastase family member 3A (74). The lowered abundance of bacterial serpin after BMS is consistent with the significant reduction we observed in these host serine proteases and other digestive enzymes.

Indeed, only a few human proteins showed a significant decrease after gastric bypass (*n*=14). These were mostly related to the reduction of the gastric mucosa and the surgical rerouting of bile and pancreatic secretions (9 out of 14). Defining a specific combination of host and microbial proteins affected by biliopancreatic fluid rerouting and detectable in fecal samples deserves further investigation. Once validated, this knowledge holds the promise for prompting the evaluation and treatment of the adverse effects of biliopancreatic fluid diversion, specifically maldigestion and abdominal complaints, which are caused by exocrine pancreatic insufficiency in a significant proportion of patients (75).

While gastric and pancreatic secreted proteins were reduced, a larger number of host proteins were significantly more abundant after BMS. These changes are consistent with the enrichment of several functional pathways, including neutrophil degranulation, glycolysis, muscle contraction, and the immune system. Interestingly, the most significant variation concerned lactotransferrin (also known as lactoferrin), an iron-binding glycoprotein with established antimicrobial and immunomodulatory activities (76–78). Other intestinal proteins showing marked increases in relative abundance after BMS are well known for their roles in mucosal homeostasis (lipocalin-1, meprin A, alpha-2-macroglobulin, galectin-3 binding protein, ACE2, and leukocyte elastase inhibitor), intestinal inflammation (neutrophil effectors myeloperoxidase and neutrophil elastase), and antimicrobial defense (S100-A8 and REG3A). The increased abundance of the metalloprotease meprin A at T1 is noteworthy, as it is known to be highly expressed in the healthy gut to regulate host-microbiome interactions, but significantly reduced in inflammatory bowel disease (79, 80). Consistently, RNA-seq data have previously demonstrated that the pathways most enriched in the distal small intestine after BMS include muscle contraction, immune activation, and especially antimicrobial peptides like REG3A (81). Furthermore, REG3A has been found to regulate glucose homeostasis and insulin resistance in obese diabetic mice (82).

Together, these findings underscore the value of metaproteomics in capturing the functional dimension of GM dynamics and highlight potential microbial and host protein biomarkers that could inform the management of post-surgical outcomes, including nutritional adaptation and metabolic health.

## Data Availability

The mass spectrometry proteomics data (including peptide identification, quantification, and annotation tables) have been deposited to the ProteomeXchange Consortium via the PRIDE (83) partner repository with the data set identifier PXD065166.

## SUPPLEMENTAL MATERIAL

Supplemental Material msystems.01764-25-s0001.pdf Figures S1-S9; Tables S1-S5.

ASM does not own the copyrights to Supplemental Material that may be linked to, or accessed through, an article. The authors have granted ASM a non-exclusive, world-wide license to publish the Supplemental Material files. Please contact the corresponding author directly for reuse.

## References

[1] Eisenberg D, Shikora SA, Aarts E, Aminian A, Angrisani L, Cohen RV, de Luca M, Faria SL, Goodpaster KPS, Haddad A, Himpens JM, Kow L, Kurian M, Loi K, Mahawar K, Nimeri A, O’Kane M, Papasavas PK, Ponce J, Pratt JSA, Rogers AM, Steele KE, Suter M, Kothari SN. 2023. 2022 American Society of Metabolic and Bariatric Surgery (ASMBS) and International Federation for the Surgery of Obesity and Metabolic Disorders (IFSO) Indications for Metabolic and Bariatric Surgery. Obes Surg 33:3–14. doi:10.1007/s11695-022-06332-1 36336720 PMC9834364

[2] Zhang H, DiBaise JK, Zuccolo A, Kudrna D, Braidotti M, Yu Y, Parameswaran P, Crowell MD, Wing R, Rittmann BE, Krajmalnik-Brown R. 2009. Human gut microbiota in obesity and after gastric bypass. Proc Natl Acad Sci USA 106:2365–2370. doi:10.1073/pnas.0812600106 19164560 PMC2629490

[3] Mechanick JI, Apovian C, Brethauer S, Garvey WT, Joffe AM, Kim J, Kushner RF, Lindquist R, Pessah-Pollack R, Seger J, Urman RD, Adams S, Cleek JB, Correa R, Figaro MK, Flanders K, Grams J, Hurley DL, Kothari S, Still CD. 2020. Clinical practice guidelines for the perioperative nutrition, metabolic, and nonsurgical support of patients undergoing bariatric procedures – 2019 update. Surg Obes Relat Dis 16:175–247. doi:10.1016/j.soard.2019.10.025 31917200

[4] Luijten JCHBM, Vugts G, Nieuwenhuijzen GAP, Luyer MDP. 2019. The importance of the microbiome in bariatric surgery: a systematic review. Obes Surg 29:2338–2349. doi:10.1007/s11695-019-03863-y 30982169

[5] Amin U, Huang D, Dhir A, Shindler AE, Franks AE, Thomas CJ. 2024. Effects of gastric bypass bariatric surgery on gut microbiota in patients with morbid obesity. Gut Microbes 16:2427312. doi:10.1080/19490976.2024.2427312 39551972 PMC11581163

[6] Lazaro A, Tiago I, Mendes J, Ribeiro J, Bernardes A, Oliveira F, Regateiro F, Caramelo F, Silva H. 2025. Sleeve gastrectomy and gastric bypass impact in patient's metabolic, gut microbiome, and immuno-inflammatory profiles—a comparative study. Obes Surg 35:733–745. doi:10.1007/s11695-025-07708-9 39870942 PMC11906558

[7] Davies NK, O’Sullivan JM, Plank LD, Murphy R. 2019. Altered gut microbiome after bariatric surgery and its association with metabolic benefits: a systematic review. Surg Obes Relat Dis 15:656–665. doi:10.1016/j.soard.2019.01.033 30824335

[8] Liou AP, Paziuk M, Luevano JM, Machineni S, Turnbaugh PJ, Kaplan LM. 2013. Conserved shifts in the gut microbiota due to gastric bypass reduce host weight and adiposity. Sci Transl Med 5:178ra41. doi:10.1126/scitranslmed.3005687 PMC3652229 23536013

[9] Hamamah S, Hajnal A, Covasa M. 2024. Influence of bariatric surgery on gut microbiota composition and its implication on brain and peripheral targets. Nutrients 16:1071. doi:10.3390/nu16071071 38613104 PMC11013759

[10] Penney NC, Yeung DKT, Garcia-Perez I, Posma JM, Kopytek A, Garratt B, Ashrafian H, Frost G, Marchesi JR, Purkayastha S, Hoyles L, Darzi A, Holmes E. 2022. Multi-omic phenotyping reveals host-microbe responses to bariatric surgery, glycaemic control and obesity. Commun Med 2:127. doi:10.1038/s43856-022-00185-6 36217535 PMC9546886

[11] Palleja A, Kashani A, Allin KH, Nielsen T, Zhang C, Li Y, Brach T, Liang S, Feng Q, Jørgensen NB, Bojsen-Møller KN, Dirksen C, Burgdorf KS, Holst JJ, Madsbad S, Wang J, Pedersen O, Hansen T, Arumugam M. 2016. Roux-en-Y gastric bypass surgery of morbidly obese patients induces swift and persistent changes of the individual gut microbiota. Genome Med 8:67. doi:10.1186/s13073-016-0312-1 27306058 PMC4908688

[12] Dang JT, Mocanu V, Park H, Laffin M, Hotte N, Karmali S, Birch DW, Madsen KL. 2022. Roux-en-Y gastric bypass and sleeve gastrectomy induce substantial and persistent changes in microbial communities and metabolic pathways. Gut Microbes 14:2050636. doi:10.1080/19490976.2022.2050636 35316158 PMC8942407

[13] Li JV, Ashrafian H, Sarafian M, Homola D, Rushton L, Barker G, Cabrera PM, Lewis MR, Darzi A, Lin E, Gletsu-Miller NA, Atkin SL, Sathyapalan T, Gooderham NJ, Nicholson JK, Marchesi JR, Athanasiou T, Holmes E. 2021. Roux-en-Y gastric bypass-induced bacterial perturbation contributes to altered host-bacterial co-metabolic phenotype. Microbiome 9:139. doi:10.1186/s40168-021-01086-x 34127058 PMC8201742

[14] Ilhan ZE, DiBaise JK, Isern NG, Hoyt DW, Marcus AK, Kang D-W, Crowell MD, Rittmann BE, Krajmalnik-Brown R. 2017. Distinctive microbiomes and metabolites linked with weight loss after gastric bypass, but not gastric banding. ISME J 11:2047–2058. doi:10.1038/ismej.2017.71 28548658 PMC5563958

[15] Sanchez-Carrillo S, Ciordia S, Rojo D, Zubeldia-Varela E, Méndez-García C, Martínez-Martínez M, Barbas C, Ruiz-Ruiz S, Moya A, Garriga M, Salazar N, Botella-Carretero JI, Vega-Piñero B, de Los Reyes-Gavilán CG, Del Campo R, Ferrer M. 2021. A body weight loss- and health-promoting gut microbiota is established after bariatric surgery in individuals with severe obesity. J Pharm Biomed Anal 193:113747. doi:10.1016/j.jpba.2020.113747 33217711

[16] Van Den Bossche T, Arntzen MØ, Becher D, Benndorf D, Eijsink VGH, Henry C, Jagtap PD, Jehmlich N, Juste C, Kunath BJ, Mesuere B, Muth T, Pope PB, Seifert J, Tanca A, Uzzau S, Wilmes P, Hettich RL, Armengaud J. 2021. The Metaproteomics Initiative: a coordinated approach for propelling the functional characterization of microbiomes. Microbiome 9:243. doi:10.1186/s40168-021-01176-w 34930457 PMC8690404

[17] De Maio F, Boru CE, Velotti N, Capoccia D, Santarelli G, Verrastro O, Bianco DM, Capaldo B, Sanguinetti M, Musella M, Raffaelli M, Leonetti F, Delogu G, Silecchia G. 2024. Short-term gut microbiota’s shift after laparoscopic Roux-en-Y vs one anastomosis gastric bypass: results of a multicenter randomized control trial. Surg Endosc 38:6643–6656. doi:10.1007/s00464-024-11154-6 39294316 PMC11525425

[18] De Maio F, Boru CE, Avallone M, Velotti N, Bianco DM, Capoccia D, Greco F, Guarisco G, Nogara M, Sanguinetti M, Verrastro O, Capaldo B, Musella M, Raffaelli M, Delogu G, Silecchia G, Leonetti F. 2021. Characterization of gut microbiota in patients with metabolic syndrome candidates for bariatric/metabolic surgery: preliminary findings of a multi-center prospective study. Diabetes Res Clin Pract 180:109079. doi:10.1016/j.diabres.2021.109079 34599974

[19] Bettini S, Belligoli A, Fabris R, Busetto L. 2020. Diet approach before and after bariatric surgery. Rev Endocr Metab Disord 21:297–306. doi:10.1007/s11154-020-09571-8 32734395 PMC7455579

[20] Schulz KF, Altman DG, Moher D, CONSORT Group. 2010. CONSORT 2010 statement: updated guidelines for reporting parallel group randomised trials. BMC Med 8:18. doi:10.1186/1741-7015-8-18 20334633 PMC2860339

[21] Wiśniewski JR, Zougman A, Nagaraj N, Mann M. 2009. Universal sample preparation method for proteome analysis. Nat Methods 6:359–362. doi:10.1038/nmeth.1322 19377485

[22] Tanca A, Palomba A. 2024. Metaproteomic analysis of fecal samples from human subjects and rodent models, p 115–125. In Salerno C (ed), Methods in molecular biology. Springer US, New York, NY. 10.1007/978-1-0716-3910-8_11 38941019

[23] Palomba A, Tanca A, Abbondio M, Sau R, Serra M, Marongiu F, Fraumene C, Pagnozzi D, Laconi E, Uzzau S. 2021. Time-restricted feeding induces Lactobacillus- and Akkermansia-specific functional changes in the rat fecal microbiota. NPJ Biofilms Microbiomes 7:85. doi:10.1038/s41522-021-00256-x 34862421 PMC8642412

[24] Abbondio M, Tanca A, De Diego L, Sau R, Bibbò S, Pes GM, Dore MP, Uzzau S. 2023. Metaproteomic assessment of gut microbial and host functional perturbations in Helicobacter pylori-infected patients subjected to an antimicrobial protocol. Gut Microbes 15:2291170. doi:10.1080/19490976.2023.2291170 38063474 PMC10730194

[25] Li J, Jia H, Cai X, Zhong H, Feng Q, Sunagawa S, Arumugam M, Kultima JR, Prifti E, Nielsen T, et al.. 2014. An integrated catalog of reference genes in the human gut microbiome. Nat Biotechnol 32:834–841. doi:10.1038/nbt.2942 24997786

[26] Verschaffelt P, Van Den Bossche T, Martens L, Dawyndt P, Mesuere B. 2021. Unipept desktop: a faster, more powerful metaproteomics results analysis tool. J Proteome Res 20:2005–2009. doi:10.1021/acs.jproteome.0c00855 33401902

[27] Cantalapiedra CP, Hernández-Plaza A, Letunic I, Bork P, Huerta-Cepas J. 2021. eggNOG-mapper v2: functional annotation, orthology assignments, and domain prediction at the metagenomic scale. Mol Biol Evol 38:5825–5829. doi:10.1093/molbev/msab293 34597405 PMC8662613

[28] Galperin MY, Wolf YI, Makarova KS, Vera Alvarez R, Landsman D, Koonin EV. 2021. COG database update: focus on microbial diversity, model organisms, and widespread pathogens. Nucleic Acids Res 49:D274–D281. doi:10.1093/nar/gkaa1018 33167031 PMC7778934

[29] Kanehisa M, Furumichi M, Sato Y, Kawashima M, Ishiguro-Watanabe M. 2023. KEGG for taxonomy-based analysis of pathways and genomes. Nucleic Acids Res 51:D587–D592. doi:10.1093/nar/gkac963 36300620 PMC9825424

[30] Porcheddu M, Abbondio M, De Diego L, Uzzau S, Tanca A. 2023. Meta4P: a user-friendly tool to parse label-free quantitative metaproteomic data and taxonomic/functional annotations. J Proteome Res 22:2109–2113. doi:10.1021/acs.jproteome.2c00803 37116187 PMC10243107

[31] Tyanova S, Temu T, Sinitcyn P, Carlson A, Hein MY, Geiger T, Mann M, Cox J. 2016. The Perseus computational platform for comprehensive analysis of (prote)omics data. Nat Methods 13:731–740. doi:10.1038/nmeth.3901 27348712

[32] Benjamini Y, Hochberg Y. 1995. Controlling the false discovery rate: a practical and powerful approach to multiple testing. J R Stat Soc Ser B 57:289–300. doi:10.1111/j.2517-6161.1995.tb02031.x

[33] Szklarczyk D, Kirsch R, Koutrouli M, Nastou K, Mehryary F, Hachilif R, Gable AL, Fang T, Doncheva NT, Pyysalo S, Bork P, Jensen LJ, von Mering C. 2023. The STRING database in 2023: protein–protein association networks and functional enrichment analyses for any sequenced genome of interest. Nucleic Acids Res 51:D638–D646. doi:10.1093/nar/gkac1000 36370105 PMC9825434

[34] Milacic M, Beavers D, Conley P, Gong C, Gillespie M, Griss J, Haw R, Jassal B, Matthews L, May B, Petryszak R, Ragueneau E, Rothfels K, Sevilla C, Shamovsky V, Stephan R, Tiwari K, Varusai T, Weiser J, Wright A, Wu G, Stein L, Hermjakob H, D’Eustachio P. 2024. The reactome pathway knowledgebase 2024. Nucleic Acids Res 52:D672–D678. doi:10.1093/nar/gkad1025 37941124 PMC10767911

[35] Tanca A, Abbondio M, Fiorito G, Pira G, Sau R, Manca A, Muroni MR, Porcu A, Scanu AM, Cossu-Rocca P, De Miglio MR, Uzzau S. 2022. Metaproteomic profile of the colonic luminal microbiota from patients with colon cancer. Front Microbiol 13:869523. doi:10.3389/fmicb.2022.869523 35495697 PMC9048685

[36] Tanca A, Palomba A, Fiorito G, Abbondio M, Pagnozzi D, Uzzau S. 2024. Metaproteomic portrait of the healthy human gut microbiota. NPJ Biofilms Microbiomes 10:54. doi:10.1038/s41522-024-00526-4 38944645 PMC11214629

[37] Kleiner M, Thorson E, Sharp CE, Dong X, Liu D, Li C, Strous M. 2017. Assessing species biomass contributions in microbial communities via metaproteomics. Nat Commun 8:1558. doi:10.1038/s41467-017-01544-x 29146960 PMC5691128

[38] Wu Q, Ning Z, Zhang A, Zhang X, Sun Z, Figeys D. 2025. Operational taxon-function framework in MetaX: unveiling taxonomic and functional associations in metaproteomics. Anal Chem 97:9739–9747. doi:10.1021/acs.analchem.4c06645 40314762

[39] Sun Z, Ning Z, Wu Q, Li L, Doxey AC, Figeys D. 2025. Peptide abundance correlations in metaproteomics enhance taxonomic and functional analysis of the human gut microbiome. NPJ Biofilms Microbiomes 11:166. doi:10.1038/s41522-025-00801-y 40830110 PMC12365196

[40] Kolmeder CA, de Been M, Nikkilä J, Ritamo I, Mättö J, Valmu L, Salojärvi J, Palva A, Salonen A, de Vos WM. 2012. Comparative metaproteomics and diversity analysis of human intestinal microbiota testifies for its temporal stability and expression of core functions. PLoS One 7:e29913. doi:10.1371/journal.pone.0029913 22279554 PMC3261163

[41] Kolmeder CA, Salojärvi J, Ritari J, de Been M, Raes J, Falony G, Vieira-Silva S, Kekkonen RA, Corthals GL, Palva A, Salonen A, de Vos WM. 2016. Faecal metaproteomic analysis reveals a personalized and stable functional microbiome and limited effects of a probiotic intervention in adults. PLoS One 11:e0153294. doi:10.1371/journal.pone.0153294 27070903 PMC4829149

[42] Blakeley-Ruiz JA, Erickson AR, Cantarel BL, Xiong W, Adams R, Jansson JK, Fraser CM, Hettich RL. 2019. Metaproteomics reveals persistent and phylum-redundant metabolic functional stability in adult human gut microbiomes of Crohn’s remission patients despite temporal variations in microbial taxa, genomes, and proteomes. Microbiome 7:18. doi:10.1186/s40168-019-0631-8 30744677 PMC6371617

[43] Aron-Wisnewsky J, Prifti E, Belda E, Ichou F, Kayser BD, Dao MC, Verger EO, Hedjazi L, Bouillot JL, Chevallier JM, Pons N, Le Chatelier E, Levenez F, Ehrlich SD, Dore J, Zucker JD, Clément K. 2019. Major microbiota dysbiosis in severe obesity: fate after bariatric surgery. Gut 68:70–82. doi:10.1136/gutjnl-2018-316103 29899081 PMC7143256

[44] Liu R, Hong J, Xu X, Feng Q, Zhang D, Gu Y, Shi J, Zhao S, Liu W, Wang X, et al.. 2017. Gut microbiome and serum metabolome alterations in obesity and after weight-loss intervention. Nat Med 23:859–868. doi:10.1038/nm.4358 28628112

[45] Shen N, Caixàs A, Ahlers M, Patel K, Gao Z, Dutia R, Blaser MJ, Clemente JC, Laferrère B. 2019. Longitudinal changes of microbiome composition and microbial metabolomics after surgical weight loss in individuals with obesity. Surg Obes Relat Dis 15:1367–1373. doi:10.1016/j.soard.2019.05.038 31296445 PMC6722012

[46] Griffin NW, Ahern PP, Cheng J, Heath AC, Ilkayeva O, Newgard CB, Fontana L, Gordon JI. 2017. Prior dietary practices and connections to a human gut microbial metacommunity alter responses to diet interventions. Cell Host Microbe 21:84–96. doi:10.1016/j.chom.2016.12.006 28041931 PMC5234936

[47] Debédat J, Clément K, Aron-Wisnewsky J. 2019. Gut microbiota dysbiosis in human obesity: impact of bariatric surgery. Curr Obes Rep 8:229–242. doi:10.1007/s13679-019-00351-3 31197613

[48] Zambrano AK, Paz-Cruz E, Ruiz-Pozo VA, Cadena-Ullauri S, Tamayo-Trujillo R, Guevara-Ramírez P, Zambrano-Villacres R, Simancas-Racines D. 2024. Microbiota dynamics preceding bariatric surgery as obesity treatment: a comprehensive review. Front Nutr 11:1393182. doi:10.3389/fnut.2024.1393182 38633602 PMC11021787

[49] Graessler J, Qin Y, Zhong H, Zhang J, Licinio J, Wong ML, Xu A, Chavakis T, Bornstein AB, Ehrhart-Bornstein M, Lamounier-Zepter V, Lohmann T, Wolf T, Bornstein SR. 2013. Metagenomic sequencing of the human gut microbiome before and after bariatric surgery in obese patients with type 2 diabetes: correlation with inflammatory and metabolic parameters. Pharmacogenomics J 13:514–522. doi:10.1038/tpj.2012.43 23032991

[50] Desai MS, Seekatz AM, Koropatkin NM, Kamada N, Hickey CA, Wolter M, Pudlo NA, Kitamoto S, Terrapon N, Muller A, Young VB, Henrissat B, Wilmes P, Stappenbeck TS, Núñez G, Martens EC. 2016. A dietary fiber-deprived gut microbiota degrades the colonic mucus barrier and enhances pathogen susceptibility. Cell 167:1339–1353. doi:10.1016/j.cell.2016.10.043 27863247 PMC5131798

[51] Tremaroli V, Karlsson F, Werling M, Ståhlman M, Kovatcheva-Datchary P, Olbers T, Fändriks L, le Roux CW, Nielsen J, Bäckhed F. 2015. Roux-en-Y gastric bypass and vertical banded gastroplasty induce long-term changes on the human gut microbiome contributing to fat mass regulation. Cell Metab 22:228–238. doi:10.1016/j.cmet.2015.07.009 26244932 PMC4537510

[52] Egland PG, Palmer RJ, Kolenbrander PE. 2004. Interspecies communication in Streptococcus gordonii-Veillonella atypica biofilms: signaling in flow conditions requires juxtaposition. Proc Natl Acad Sci USA 101:16917–16922. doi:10.1073/pnas.0407457101 15546975 PMC534724

[53] Kastl AJ, Terry NA, Wu GD, Albenberg LG. 2020. The structure and function of the human small intestinal microbiota: current understanding and future directions. Cell Mol Gastroenterol Hepatol 9:33–45. doi:10.1016/j.jcmgh.2019.07.006 31344510 PMC6881639

[54] Choi S-H, Kim J-S, Park J-E, Lee KC, Eom MK, Oh BS, Yu SY, Kang SW, Han K-I, Suh MK, Lee DH, Yoon H, Kim B-Y, Lee JH, Lee JH, Lee J-S, Park S-H. 2019. Anaerotignum faecicola sp. nov., isolated from human faeces. J Microbiol 57:1073–1078. doi:10.1007/s12275-019-9268-3 31680219

[55] Herz CT, Kulterer OC, Prager M, Marculescu R, Prager G, Kautzky-Willer A, Hacker M, Trajanoski S, Köfeler HC, Gallé B, Haug AR, Berry D, Kiefer FW. 2025. Bariatric surgery promotes recruitment of brown fat linked to alterations in the gut microbiota. Eur J Endocrinol 192:603–611. doi:10.1093/ejendo/lvaf081 40366070

[56] Chambers ES, Preston T, Frost G, Morrison DJ. 2018. Role of gut microbiota-generated short-chain fatty acids in metabolic and cardiovascular health. Curr Nutr Rep 7:198–206. doi:10.1007/s13668-018-0248-8 30264354 PMC6244749

[57] Park KC, Crump NT, Louwman N, Krywawych S, Cheong YJ, Vendrell I, Gill EK, Gunadasa-Rohling M, Ford KL, Hauton D, et al.. 2023. Disrupted propionate metabolism evokes transcriptional changes in the heart by increasing histone acetylation and propionylation. Nat Cardiovasc Res 2:1221–1245. doi:10.1038/s44161-023-00365-0 38500966 PMC7615744

[58] Al-Najim W, Docherty NG, le Roux CW. 2018. Food intake and eating behavior after bariatric surgery. Physiol Rev 98:1113–1141. doi:10.1152/physrev.00021.2017 29717927

[59] Browning MG, Pessoa BM, Khoraki J, Campos GM. 2019. Changes in bile acid metabolism, transport, and signaling as central drivers for metabolic improvements after bariatric surgery. Curr Obes Rep 8:175–184. doi:10.1007/s13679-019-00334-4 30847736

[60] Hu H, Shao W, Liu Q, Liu N, Wang Q, Xu J, Zhang X, Weng Z, Lu Q, Jiao L, Chen C, Sun H, Jiang Z, Zhang X, Gu A. 2022. Gut microbiota promotes cholesterol gallstone formation by modulating bile acid composition and biliary cholesterol secretion. Nat Commun 13:252. doi:10.1038/s41467-021-27758-8 35017486 PMC8752841

[61] Anveden Å, Peltonen M, Näslund I, Torgerson J, Carlsson LMS. 2020. Long-term incidence of gallstone disease after bariatric surgery: results from the nonrandomized controlled Swedish Obese Subjects study. Surg Obes Relat Dis 16:1474–1482. doi:10.1016/j.soard.2020.05.025 32654897

[62] Andoh A, Nishida A, Takahashi K, Inatomi O, Imaeda H, Bamba S, Kito K, Sugimoto M, Kobayashi T. 2016. Comparison of the gut microbial community between obese and lean peoples using 16S gene sequencing in a Japanese population. J Clin Biochem Nutr 59:65–70. doi:10.3164/jcbn.15-152 27499582 PMC4933688

[63] Borgo F, Verduci E, Riva A, Lassandro C, Riva E, Morace G, Borghi E. 2017. Relative abundance in bacterial and fungal gut microbes in obese children: a case control study. Child Obes 13:78–84. doi:10.1089/chi.2015.0194 27007700

[64] Fernandes J, Su W, Rahat-Rozenbloom S, Wolever TMS, Comelli EM. 2014. Adiposity, gut microbiota and faecal short chain fatty acids are linked in adult humans. Nutr Diabetes 4:e121. doi:10.1038/nutd.2014.23 24979150 PMC4079931

[65] Balamurugan R, George G, Kabeerdoss J, Hepsiba J, Chandragunasekaran AMS, Ramakrishna BS. 2010. Quantitative differences in intestinal Faecalibacterium prausnitzii in obese Indian children. Br J Nutr 103:335–338. doi:10.1017/S0007114509992182 19849869

[66] Zöggeler T, Kavallar AM, Pollio AR, Aldrian D, Decristoforo C, Scholl-Bürgi S, Müller T, Vogel GF. 2025. Meta-analysis of shotgun sequencing of gut microbiota in obese children with MASLD or MASH. Gut Microbes 17:2508951. doi:10.1080/19490976.2025.2508951 40396204 PMC12101585

[67] De la Cuesta-Zuluaga J, Mueller N, Álvarez-Quintero R, Velásquez-Mejía E, Sierra J, Corrales-Agudelo V, Carmona J, Abad J, Escobar J. 2019. Higher fecal short-chain fatty acid levels are associated with gut microbiome dysbiosis, obesity, hypertension and cardiometabolic disease risk factors. Nutrients 11:51. doi:10.3390/nu11010051 PMC6356834 30591685

[68] Murugesan S, Ulloa-Martínez M, Martínez-Rojano H, Galván-Rodríguez FM, Miranda-Brito C, Romano MC, Piña-Escobedo A, Pizano-Zárate ML, Hoyo-Vadillo C, García-Mena J. 2015. Study of the diversity and short-chain fatty acids production by the bacterial community in overweight and obese Mexican children. Eur J Clin Microbiol Infect Dis 34:1337–1346. doi:10.1007/s10096-015-2355-4 25761741

[69] Ettehad Marvasti F, Moshiri A, Taghavi MS, Riazi S, Taati M, Sadati SF, Ghaheri A, Masoomi M, Vaziri F, Fateh A, Rohani P, Tarashi S, Masotti A, Ahmadi Badi S, Siadat SD. 2020. The first report of differences in gut microbiota composition between obese and normal weight Iranian subjects. Iran Biomed J 24:148–154. doi:10.29252/ibj.24.3.148 31952432 PMC7275621

[70] Hippe B, Remely M, Aumueller E, Pointner A, Magnet U, Haslberger AG. 2016. Faecalibacterium prausnitzii phylotypes in type two diabetic, obese, and lean control subjects. Benef Microbes 7:511–517. doi:10.3920/BM2015.0075 27048834

[71] Schmid A, Liebisch G, Burkhardt R, Belikan H, Köhler S, Steger D, Schweitzer L, Pons-Kühnemann J, Karrasch T, Schäffler A. 2024. Dynamics of the human bile acid metabolome during weight loss. Sci Rep 14:25743. doi:10.1038/s41598-024-75831-1 39468179 PMC11519931

[72] Peng YL, Wang SH, Zhang YL, Chen MY, He K, Li Q, Huang WH, Zhang W. 2024. Effects of bile acids on the growth, composition and metabolism of gut bacteria. NPJ Biofilms Microbiomes 10:112. doi:10.1038/s41522-024-00566-w 39438471 PMC11496524

[73] Haange S-B, Jehmlich N, Krügel U, Hintschich C, Wehrmann D, Hankir M, Seyfried F, Froment J, Hübschmann T, Müller S, Wissenbach DK, Kang K, Buettner C, Panagiotou G, Noll M, Rolle-Kampczyk U, Fenske W, von Bergen M. 2020. Gastric bypass surgery in a rat model alters the community structure and functional composition of the intestinal microbiota independently of weight loss. Microbiome 8:13. doi:10.1186/s40168-020-0788-1 32033593 PMC7007695

[74] Mariaule V, Kriaa A, Soussou S, Rhimi S, Boudaya H, Hernandez J, Maguin E, Lesner A, Rhimi M. 2021. Digestive inflammation: role of proteolytic dysregulation. Int J Mol Sci 22:2817. doi:10.3390/ijms22062817 33802197 PMC7999743

[75] Moore HN, Chirco AR, Plescia T, Ahmed S, Jachniewicz B, Rajasekar G, Ali MR, Lyo V. 2023. Exocrine pancreatic insufficiency after bariatric surgery: a bariatric surgery center of excellence experience. Surg Endosc 37:1466–1475. doi:10.1007/s00464-022-09388-3 35768735

[76] Kanyshkova TG, Buneva VN, Nevinsky GA. 2001. Lactoferrin and its biological functions. Biochemistry (Moscow) 66:1–7. doi:10.1023/A:1002817226110 11240386

[77] Jenssen H, Hancock REW. 2009. Antimicrobial properties of lactoferrin. Biochimie 91:19–29. doi:10.1016/j.biochi.2008.05.015 18573312

[78] Berlutti F, Pantanella F, Natalizi T, Frioni A, Paesano R, Polimeni A, Valenti P. 2011. Antiviral properties of lactoferrin—a natural immunity molecule. Molecules 16:6992–7018. doi:10.3390/molecules16086992 21847071 PMC6264778

[79] Werny L, Colmorgen C, Becker-Pauly C. 2022. Regulation of meprin metalloproteases in mucosal homeostasis. Biochim Biophys Acta Mol Cell Res 1869:119158. doi:10.1016/j.bbamcr.2021.119158 34626680

[80] Bülck C, Nyström EEL, Koudelka T, Mannbar-Frahm M, Andresen G, Radhouani M, Tran F, Scharfenberg F, Schrell F, Armbrust F, Dahlke E, Zhao B, Vervaeke A, Theilig F, Rosenstiel P, Starkl P, Rosshart SP, Fickenscher H, Tholey A, Hansson GC, Becker-Pauly C. 2023. Proteolytic processing of galectin-3 by meprin metalloproteases is crucial for host-microbiome homeostasis. Sci Adv 9:eadf4055. doi:10.1126/sciadv.adf4055 37000885 PMC10065446

[81] Shin JH, Bozadjieva-Kramer N, Shao Y, Lyons-Abbott S, Rupp AC, Sandoval DA, Seeley RJ. 2022. The gut peptide Reg3g links the small intestine microbiome to the regulation of energy balance, glucose levels, and gut function. Cell Metab 34:1765–1778. doi:10.1016/j.cmet.2022.09.024 36240758 PMC9633559

[82] Gonzalez P, Dos Santos A, Darnaud M, Moniaux N, Rapoud D, Lacoste C, Nguyen TS, Moullé VS, Deshayes A, Amouyal G, Amouyal P, Bréchot C, Cruciani-Guglielmacci C, Andréelli F, Magnan C, Faivre J. 2023. Antimicrobial protein REG3A regulates glucose homeostasis and insulin resistance in obese diabetic mice. Commun Biol 6:269. doi:10.1038/s42003-023-04616-5 36918710 PMC10015038

[83] Perez-Riverol Y, Bai J, Bandla C, García-Seisdedos D, Hewapathirana S, Kamatchinathan S, Kundu DJ, Prakash A, Frericks-Zipper A, Eisenacher M, Walzer M, Wang S, Brazma A, Vizcaíno JA. 2022. The PRIDE database resources in 2022: a hub for mass spectrometry-based proteomics evidences. Nucleic Acids Res 50:D543–D552. doi:10.1093/nar/gkab1038 34723319 PMC8728295

