# Supplementary material for: Reshaping of the fecal proteome and metaproteome in obese patients 2 years after bariatric surgery
Source: mSystems. 2026 Jun 9;11(7):e01764-25. doi: 10.1128/msystems.01764-25 (PMC13386881; doi:10.1128/msystems.01764-25)
Supplement: Supplemental Material — Figures S1-S9; Tables S1-S5. [file msystems.01764-25-s0001.pdf]

Supplementary material

Figure S1. PCA plot based on the relative abundances of microbial taxa.
Figure S2. PCA plot based on the relative abundances of microbial KO functions.
Figure S3. PCA plot based on relative abundances of host proteins.
Figure S4. Relative abundance ranking distribution of KEGG KO functions belonging to the glycolysis pathway, as measured in this study and in a fecal metaproteome dataset from healthy individuals.
Figure S5. Relative abundance ranking distribution of KEGG KO functions belonging to carbohydrate metabolism pathways other than glycolysis, as measured in this study and in a fecal metaproteome dataset from healthy individuals.
Figure S6. Relative abundance ranking distribution of KEGG KO functions belonging to amino acid metabolism, cofactor and vitamin metabolism, and nucleotide binding and metabolism pathways, as measured in this study and in a fecal metaproteome dataset from healthy individuals.
Figure S7. Relative abundance ranking distribution of KEGG KO functions belonging to the bacterial cell wall or membranes, as measured in this study and in a fecal metaproteome dataset from healthy individuals.
Figure S8. Relative abundance ranking distribution of a selection of KEGG KO functions shown in Figure 7, as measured in this study and in a fecal metaproteome dataset from healthy individuals.
Figure S9. Pearson correlation between the relative abundance of the bacterial nitrogen fixation protein NifU measured at T1 and the relative variation of cortisol between T1 and T0.

Table S1. Main characteristics of the patients recruited in this study.
Table S2. Taxa with significantly differential abundance between T1 and T0.
Table S3. General and taxon-specific KEGG KO functions with significantly differential abundance between T1 and T0.
Table S4. Human proteins with significantly higher abundance at T1 compared to T0.
Table S5. Anthropometric and blood parameters measured in the patients recruited in this study.

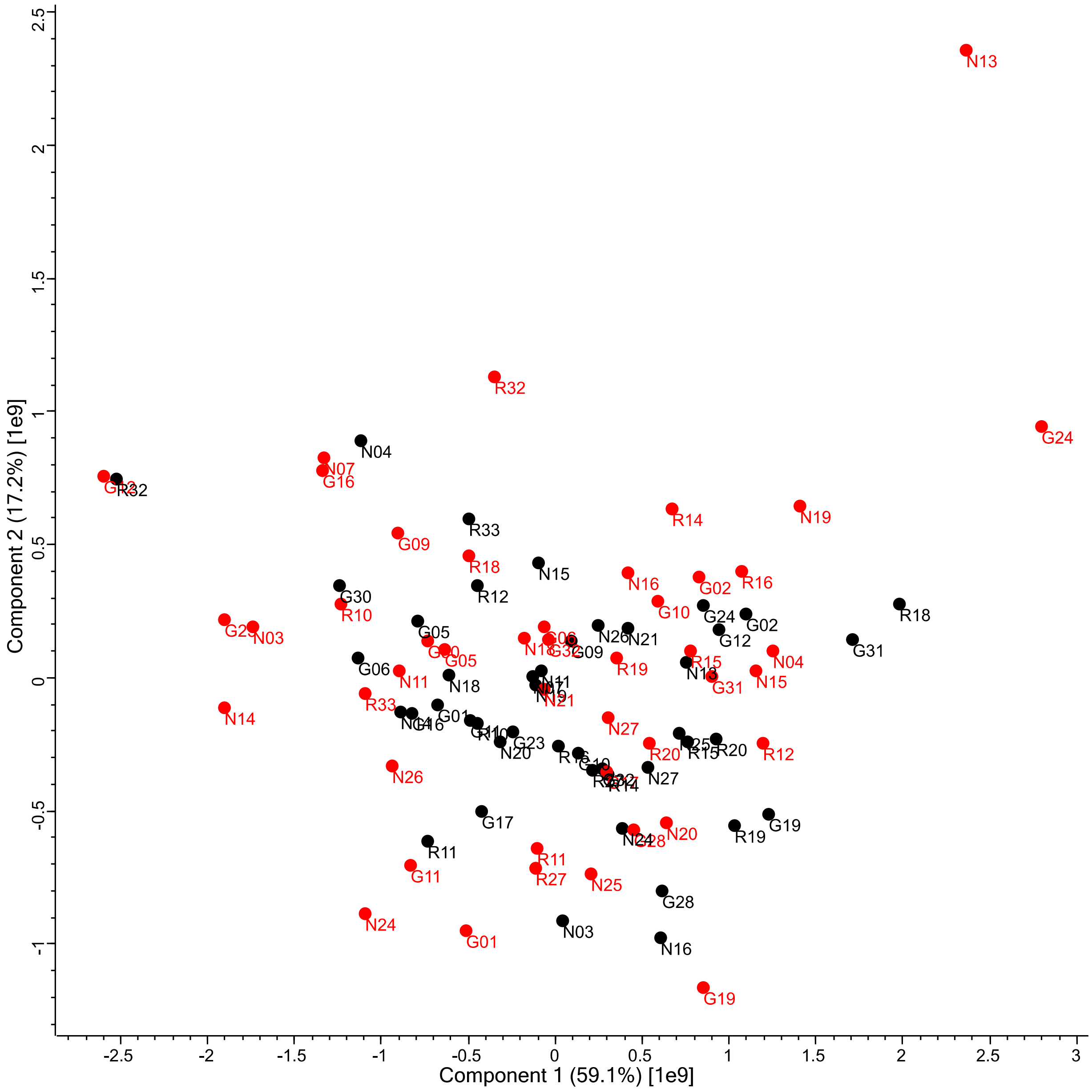

Figure S1. PCA plot based on the relative abundances of microbial taxa. Each point marks a different sample, with T0 and T1 samples colored in red and black, respectively, and the patient code given next to each point. The percentages of variation explained by the first two components are shown on the x and y axes, respectively.

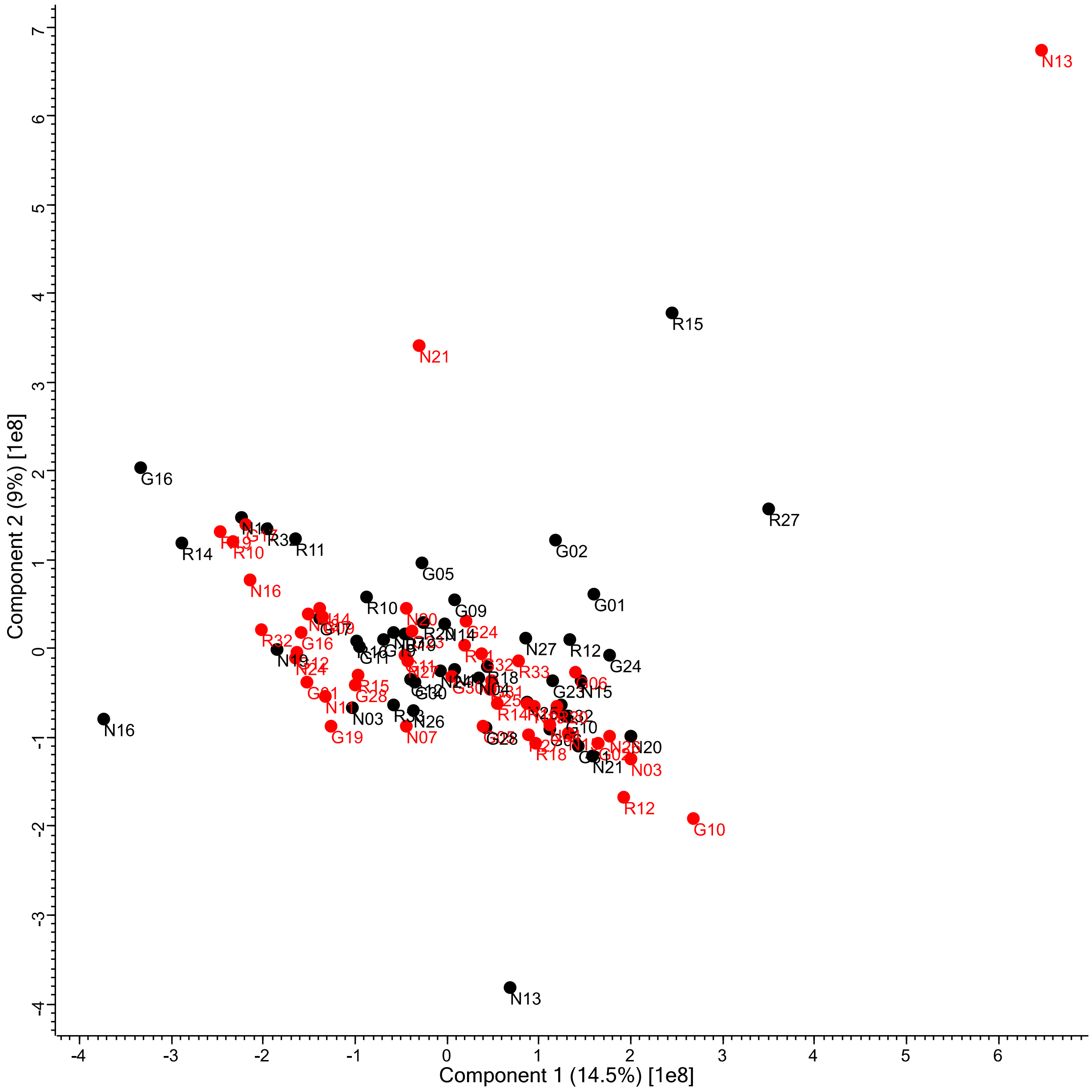

Figure S2. PCA plot based on the relative abundances of microbial KO functions. Each point marks a different sample, with T0 and T1 samples colored in red and black, respectively, and the patient code given next to each point. The percentages of variation explained by the first two components are shown on the x and y axes, respectively.

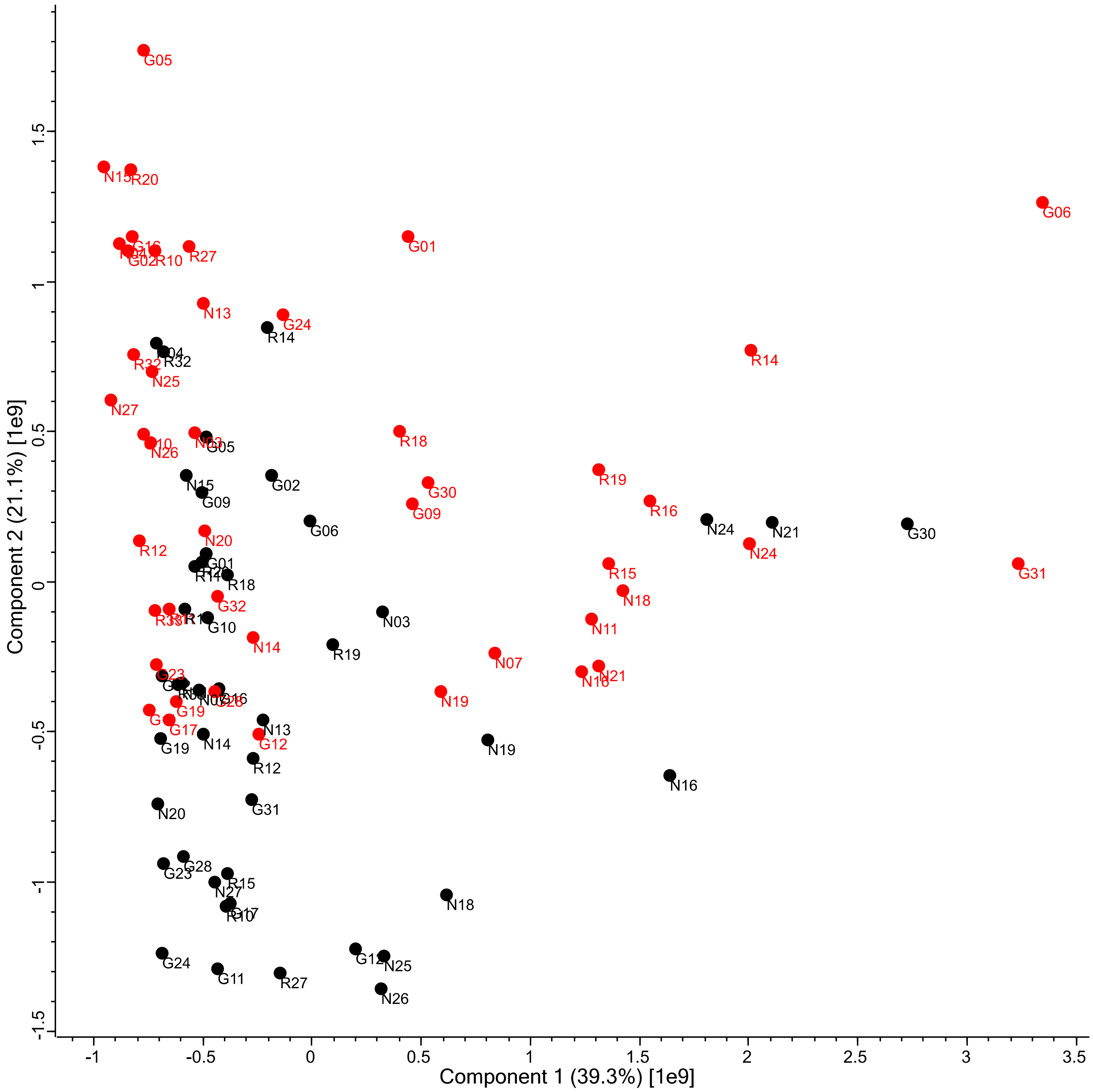

Figure S3. PCA plot based on relative abundances of host proteins. Each point marks a different sample, with T0 and T1 samples colored in red and black, respectively, and the patient code given next to each point. The percentages of variation explained by the first two components are shown on the x and y axes, respectively.

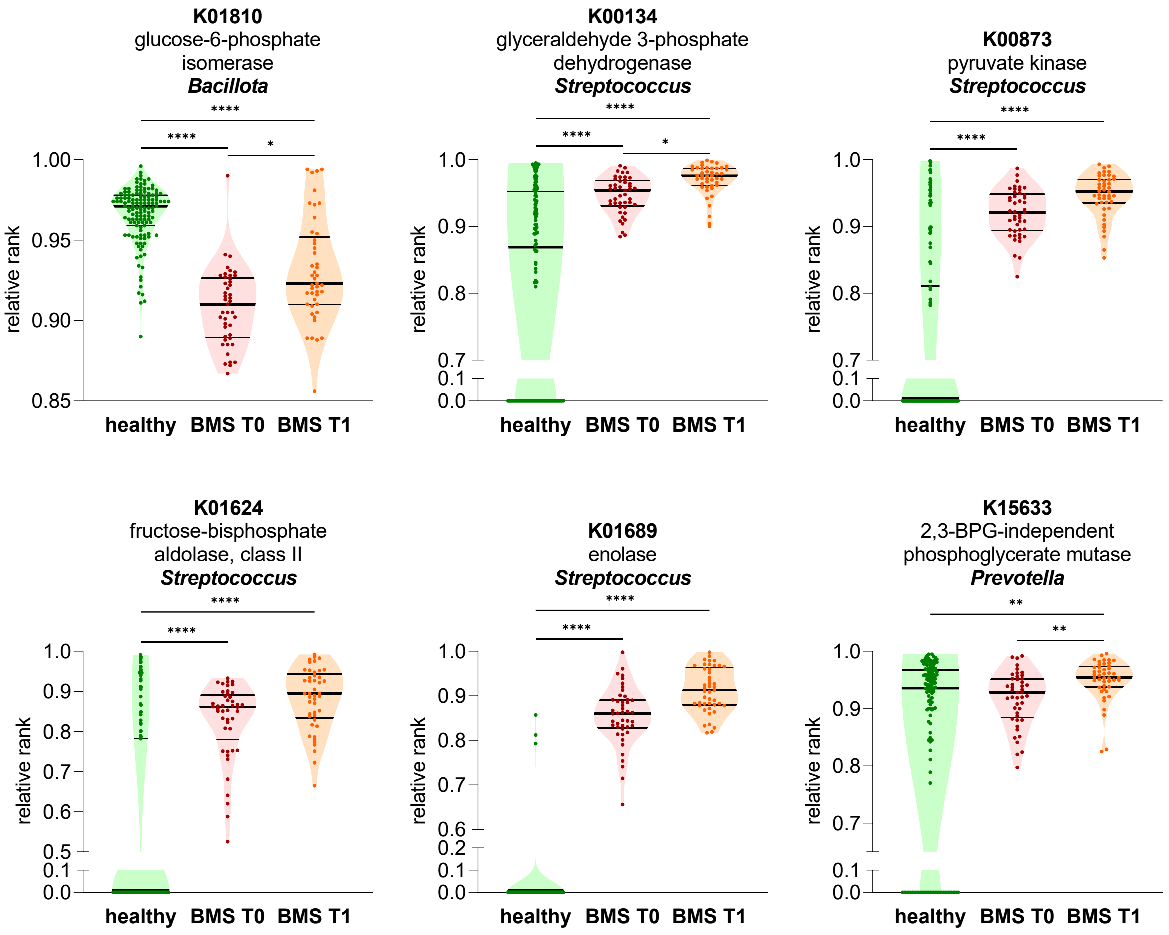

Figure S4. Relative abundance ranking distribution of taxon-specific KEGG KO functions belonging to the glycolysis pathway, as measured in this study (BMS T0 and BMS T1) and in a collection of fecal metaproteome datasets from healthy individuals (healthy). The differential KOs reported in Figure 3 and available in the "healthy" dataset are shown. Each circle represents an individual subject/patient. The horizontal thick black lines indicate the median of the distributions, while the thinner lines indicate the upper and lower quartiles. Statistical significance was calculated using a Kruskal-Wallis test with Dunn's correction for multiple comparisons (* p < 0.05; ** p < 0.01; **** p < 0.0001).

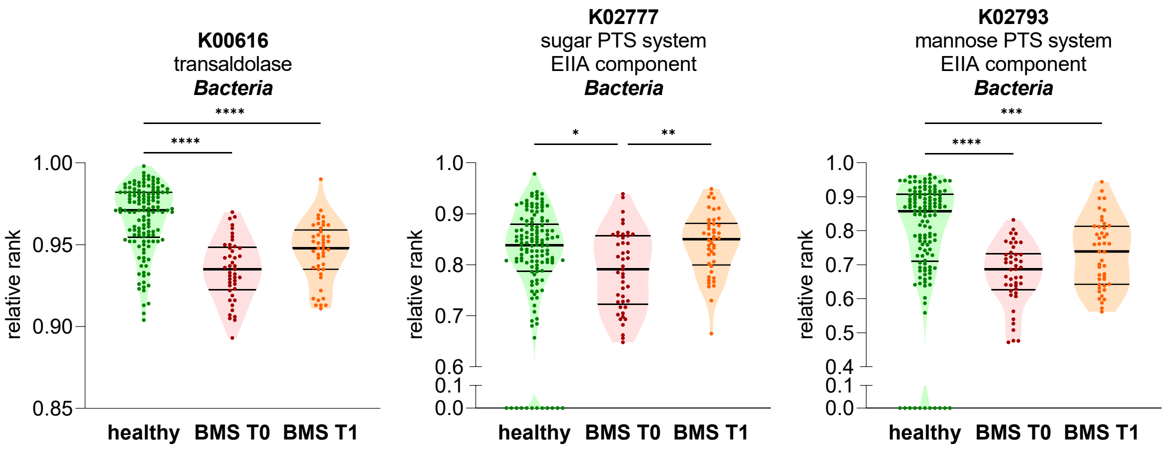

Figure S5. Relative abundance ranking distribution of KEGG KO functions belonging to carbohydrate metabolism pathways other than glycolysis, as measured in this study (BMS T0 and BMS T1) and in a collection of fecal metaproteome datasets from healthy individuals (healthy). The differential KOs reported in Figure 4 and available in the "healthy" dataset are shown. Each circle represents an individual subject/patient. The horizontal thick black lines indicate the median of the distributions, while the thinner lines indicate the upper and lower quartiles. Statistical significance was calculated using a Kruskal-Wallis test with Dunn's correction for multiple comparisons (* $p < 0.05$; ** $p < 0.01$; *** $p < 0.001$; **** $p < 0.0001$).

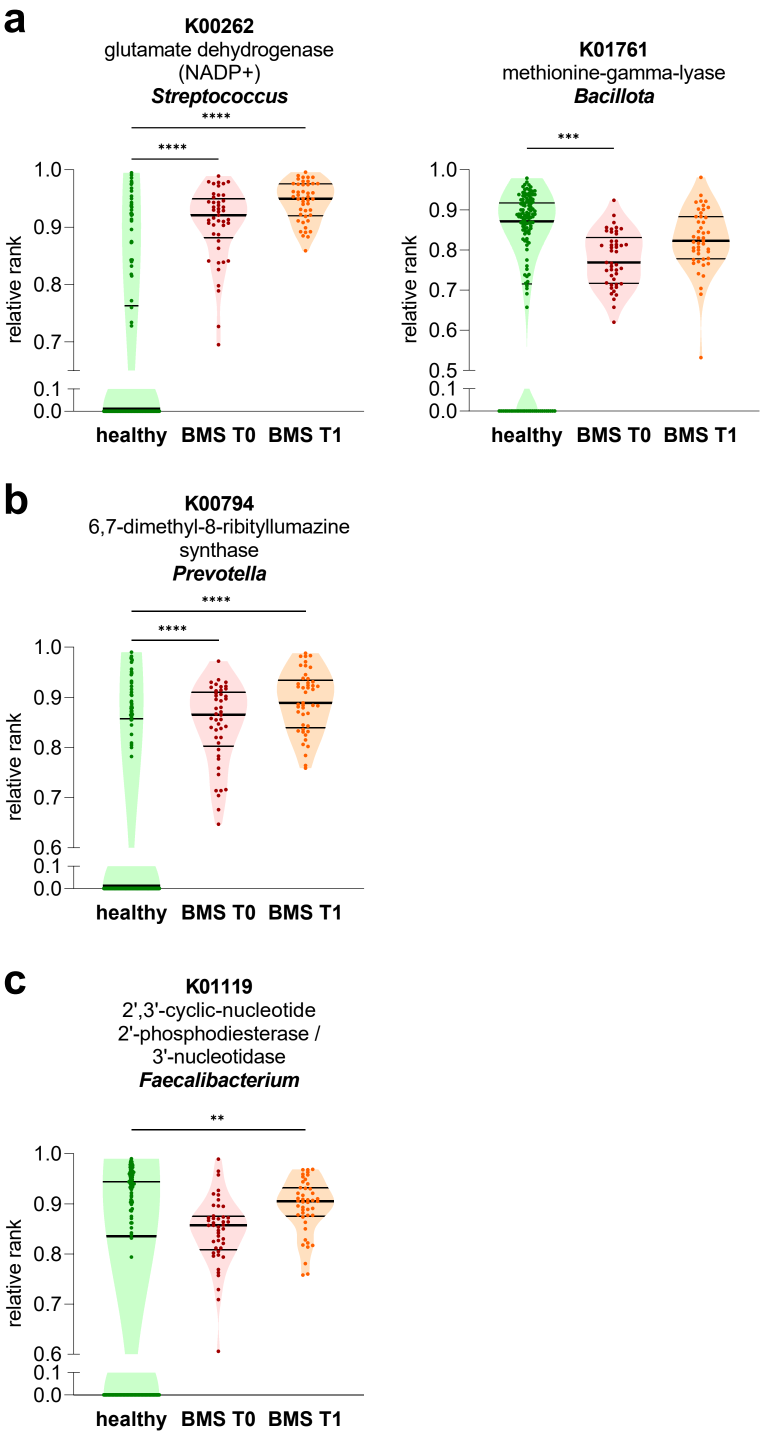

Figure S6. Relative abundance ranking distribution of taxon-specific KEGG KO functions belonging to amino acid metabolism (**a**), cofactor and vitamin metabolism (**b**), and nucleotide binding and metabolism (**c**) pathways, as measured in this study (BMS T0 and BMS T1) and in a collection of fecal metaproteome datasets from healthy individuals (healthy). The differential KOs reported in Figure 5 and available in the "healthy" dataset are shown. Each circle represents an individual subject/patient. The horizontal thick black lines indicate the median of the distributions, while the thinner lines indicate the upper and lower quartiles. Statistical significance was calculated using a Kruskal-Wallis test with Dunn's correction for multiple comparisons (** p < 0.01; *** p < 0.001; **** p < 0.0001).

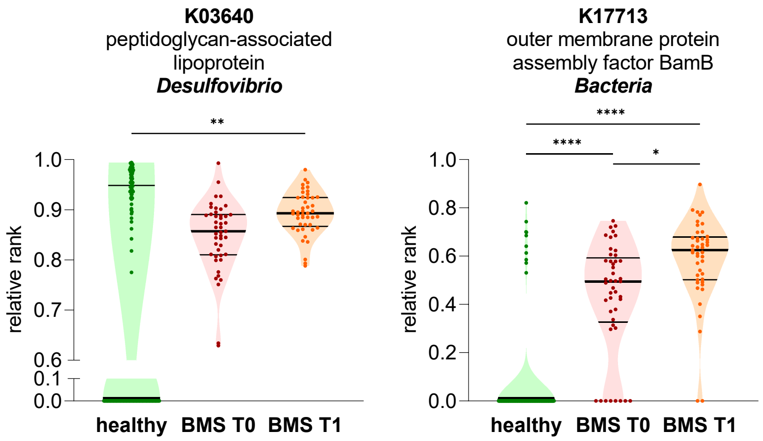

Figure S7. Relative abundance ranking distribution of KEGG KO functions belonging to the bacterial cell wall or membranes, as measured in this study (BMS T0 and BMS T1) and in a collection of fecal metaproteome datasets from healthy individuals (healthy). The differential KOs reported in Figure 6 and available in the "healthy" dataset are shown. Each circle represents an individual subject/patient. The horizontal thick black lines indicate the median of the distributions, while the thinner lines indicate the upper and lower quartiles. Statistical significance was calculated using a Kruskal-Wallis test with Dunn's correction for multiple comparisons (* $p < 0.05$; ** $p < 0.01$; **** $p < 0.0001$).

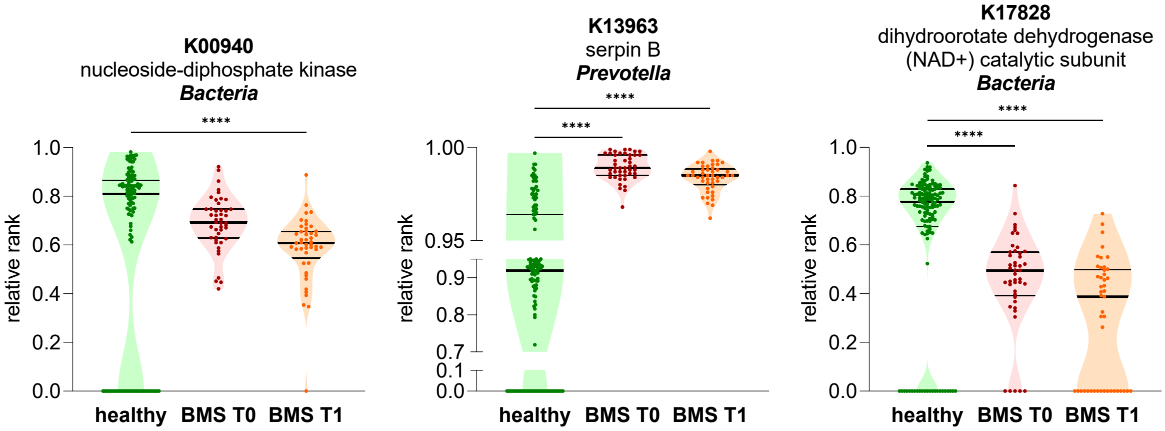

Figure S8. Relative abundance ranking distribution of a selection of KEGG KO functions, as measured in this study (BMS T0 and BMS T1) and in a collection of fecal metaproteome datasets from healthy individuals (healthy). KOs reported in Figure 7 and available in the "healthy" dataset are shown. Each circle represents an individual subject/patient. The horizontal thick black lines indicate the median of the distributions, while the thinner lines indicate the upper and lower quartiles. Statistical significance was calculated using a Kruskal-Wallis test with Dunn's correction for multiple comparisons (**** $p < 0.0001$).

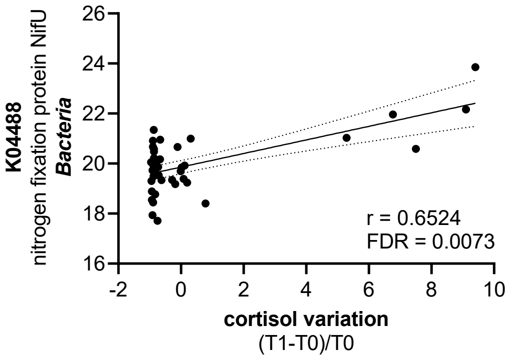

Figure S9. Scatter plot showing the Pearson correlation with linear regression lines (mean and 95% confidence interval) between the log-transformed relative abundance of the bacterial nitrogen fixation protein NifU, as measured in patients' fecal metaproteomes at T1, and the relative variation of cortisol between T1 and T0, as measured in patients' blood. The Pearson correlation coefficient (r) and the false discovery rate (FDR), calculated according to Benjamini-Hochberg's correction for multiple testing, are also shown.

Table S1. Main characteristics of the patients recruited in this study.

Sample code	Hospital	Surgery	Clinical classification	Sex	BMI T0	BMI T1	Excess BMI loss
G01	Gemelli	RYGB	obesity	female	40.79	24.34	104.18
G02	Gemelli	RYGB	pre metabolic syndrome	male	51.81	38.7	48.9
G05	Gemelli	MGB	pre metabolic syndrome	female	40.23	30.47	64.08
G06	Gemelli	MGB	obesity	male	40.4	25.72	95.32
G09	Gemelli	MGB	pre metabolic syndrome	male	49.72	29.54	81.63
G10	Gemelli	RYGB	pre metabolic syndrome	male	40.04	23.16	112.23
G11	Gemelli	MGB	pre metabolic syndrome	male	51.81	35.27	61.25
G12	Gemelli	RYGB	pre metabolic syndrome	male	43.35	28.33	82.67
G16	Gemelli	RYGB	obesity	female	45.03	30.02	75.54
G17	Gemelli	MGB	metabolic syndrome	male	48.97	35.44	56.45
G19	Gemelli	RYGB	pre metabolic syndrome	female	51.86	32.97	70.33
G23	Gemelli	RYGB	obesity	male	49.03	27.51	89.46
G24	Gemelli	RYGB	obesity	male	47.4	29.34	80.63
G28	Gemelli	MGB	obesity	female	42.24	32.32	57.54
G30	Gemelli	MGB	obesity	female	46.09	30.08	75.91
G31	Gemelli	RYGB	pre metabolic syndrome	female	40.77	26.08	93.22
G32	Gemelli	MGB	obesity	female	41.41	29.3	71.77
N03	Napoli	MGB	obesity	female	40.19	24.33	104.41
N04	Napoli	RYGB	obesity	female	47.87	30.09	77.74
N07	Napoli	RYGB	pre metabolic syndrome	female	46.61	31.12	71.68
N11	Napoli	MGB	metabolic syndrome	male	44.62	27.72	86.14
N13	Napoli	MGB	pre metabolic syndrome	female	42.3	29.72	72.72
N14	Napoli	MGB	obesity	female	40.62	24.22	104.99
N15	Napoli	MGB	pre metabolic syndrome	male	43.73	30.32	71.6
N16	Napoli	RYGB	pre metabolic syndrome	female	51.67	33.41	68.7
N18	Napoli	RYGB	obesity	female	47.78	34.08	60.14
N19	Napoli	RYGB	pre metabolic syndrome	male	51.21	22.92	107.94
N20	Napoli	MGB	pre metabolic syndrome	female	41.8	26.37	91.75
N21	Napoli	MGB	metabolic syndrome	female	42.53	27.96	83.11
N24	Napoli	MGB	pre metabolic syndrome	female	46.25	35.94	48.52
N25	Napoli	MGB	metabolic syndrome	male	49.31	30.69	76.59
N26	Napoli	MGB	pre metabolic syndrome	male	43.07	31.27	65.3
N27	Napoli	RYGB	pre metabolic syndrome	male	40.08	27.4	84.08
R10	Latina	MGB	pre metabolic syndrome	female	43.94	21.8	116.9
R11	Latina	RYGB	metabolic syndrome	female	40.98	27.59	83.79

Sample code	Hospital	Surgery	Clinical classification	Sex	BMI T0	BMI T1	Excess BMI loss
R12	Latina	MGB	pre metabolic syndrome	male	49.83	25.61	97.54
R14	Latina	RYGB	pre metabolic syndrome	female	49.68	31.99	72
R15	Latina	MGB	metabolic syndrome	female	48.7	31.76	71.48
R16	Latina	RYGB	metabolic syndrome	female	49.95	31.22	75.07
R18	Latina	RYGB	obesity	female	41.01	24.13	105.43
R19	Latina	RYGB	pre metabolic syndrome	female	43.44	29.54	75.38
R20	Latina	MGB	obesity	female	54.01	38.58	53.19
R27	Latina	MGB	obesity	female	43.26	26.04	94.3
R32	Latina	MGB	pre metabolic syndrome	male	44.98	31.14	68.33
R33	Latina	RYGB	pre metabolic syndrome	female	51.31	31.25	76.24

Table S2. Taxa with significantly differential abundance between T1 and T0. Difference values and q-values were obtained by paired sample Student's t-test followed by Benjamini and Hochberg's adjustment for multiple testing. Tests were performed separately for each taxonomic level. Taxa are presented based on their taxonomic level, and within each taxonomic level in order of decreasing difference value.

taxon	taxonomic level	taxonomic lineage	peptides	q-value	difference (T1-T0)
Verrucomicrobiota	phylum	Verrucomicrobiota	133	0.047407	0.829
Verrucomicrobiae	class	Verrucomicrobiota | Verrucomicrobiae	130	0.031238	0.926
Bacilli	class	Bacillota | Bacilli	1246	0.000372	0.539
Verrucomicrobiales	order	Verrucomicrobiota | Verrucomicrobiae | Verrucomicrobiales	130	0.024990	0.926
Lactobacillales	order	Bacillota | Bacilli | Lactobacillales	1168	0.000528	0.550
Veillonellales	order	Bacillota | Negativicutes | Veillonellales	2420	0.007249	0.368
Akkermansiaceae	family	Verrucomicrobiota | Verrucomicrobiae | Verrucomicrobiales | Akkermansiaceae	130	0.023372	0.926
Streptococcaceae	family	Bacillota | Bacilli | Lactobacillales | Streptococcaceae	598	0.000038	0.793
Veillonellaceae	family	Bacillota | Negativicutes | Veillonellales | Veillonellaceae	2420	0.007019	0.368
Oscillospiraceae	family	Bacillota | Clostridia | Eubacteriales | Oscillospiraceae	6265	0.023372	-0.209
Peptostreptococcaceae	family	Bacillota | Clostridia | Eubacteriales | Peptostreptococcaceae	103	0.000740	-0.779
Akkermansia	genus	Verrucomicrobiota | Verrucomicrobiae | Verrucomicrobiales | Akkermansiaceae | Akkermansia	130	0.032906	0.926
Anaerotignum	genus	Bacillota | Clostridia | Eubacteriales | Lachnospiraceae | Anaerotignum	16	0.006380	0.922
Streptococcus	genus	Bacillota | Bacilli | Lactobacillales | Streptococcaceae | Streptococcus	575	0.000095	0.806
Veillonella	genus	Bacillota | Negativicutes | Veillonellales | Veillonellaceae | Veillonella	461	0.000126	0.804
Desulfovibrio	genus	Pseudomonadota | Deltaproteobacteria | Desulfovibrionales | Desulfovibrionaceae | Desulfovibrio	85	0.032906	0.574
Faecalibacterium	genus	Bacillota | Clostridia | Eubacteriales | Oscillospiraceae | Faecalibacterium	2421	0.032906	-0.270
Romboutsia	genus	Bacillota | Clostridia | Eubacteriales | Peptostreptococcaceae | Romboutsia	65	0.002789	-0.765
Veillonella atypica	species	Bacillota | Negativicutes | Veillonellales | Veillonellaceae | Veillonella | Veillonella atypica	89	0.001724	0.899
Romboutsia ilealis	species	Bacillota | Clostridia | Eubacteriales | Peptostreptococcaceae | Romboutsia | Romboutsia ilealis	48	0.007071	-0.756

Table S3. KEGG KO functions with significantly differential abundance between T1 and T0. Difference values and q-values were obtained by paired sample Student's t-test followed by Benjamini and Hochberg's adjustment for multiple testing. Tests were performed separately for each taxonomic level. KO are ordered by their taxonomic specificity, and within each taxonomic level in order of decreasing difference value.

KO	KO name	taxonomic specificity	taxon name	peptides	q-value	difference (T1-T0)
K17713	outer membrane protein assembly factor BamB	none	Bacteria	4	0.03111	3.237
K06078	murein lipoprotein	none	Bacteria	24	$1.4 \cdot 10^{-7}$	1.830
K05606	methylmalonyl-CoA/ethylmalonyl-CoA epimerase	none	Bacteria	43	0.00087	1.249
K03809	NAD(P)H dehydrogenase (quinone)	none	Bacteria	15	0.00495	1.155
K03746	DNA-binding protein H-NS	none	Bacteria	11	0.02716	1.141
K11685	DNA-binding protein StpA	none	Bacteria	10	0.03063	1.136
K02793	mannose PTS system EIIA component	none	Bacteria	27	0.01814	1.010
K16076	outer membrane porin protein LC	none	Bacteria	138	0.00525	0.987
K11929	outer membrane pore protein E	none	Bacteria	174	0.00495	0.959
K09475	outer membrane pore protein C	none	Bacteria	166	0.00495	0.957
K02777	sugar PTS system EIIA component	none	Bacteria	19	0.02241	0.928
K09476	outer membrane pore protein F	none	Bacteria	187	0.00495	0.917
K14062	outer membrane protein N	none	Bacteria	187	0.00495	0.917
K02794	mannose PTS system EIIAB component	none	Bacteria	33	0.02260	0.870
K00096	glycerol-1-phosphate dehydrogenase [NAD(P)+]	none	Bacteria	23	0.03185	0.833
K22430	caffeyl-CoA reductase-Etf complex subunit CarC	none	Bacteria	9	0.03234	0.831
K00005	glycerol dehydrogenase	none	Bacteria	36	0.01814	0.830
K00821	acetylornithine/N-succinyldiaminopimelate aminotransferase	none	Bacteria	73	0.02241	0.685
K00656	formate C-acetyltransferase	none	Bacteria	272	0.01686	0.646
K03339	6-phospho-5-dehydro-2-deoxy-D-gluconate aldolase	none	Bacteria	41	0.01160	0.618
K03286	OmpA-OmpF porin, OOP family	none	Bacteria	292	0.04469	0.535
K00616	transaldolase	none	Bacteria	118	0.03063	0.446
K02110	F-type H+-transporting ATPase subunit c	none	Bacteria	63	0.02037	-0.737
K06334	spore coat protein JC	none	Bacteria	42	0.03239	-0.783
K13963	serpin B	none	Bacteria	145	0.00763	-0.807
K00940	nucleoside-diphosphate kinase	none	Bacteria	14	0.04352	-1.449
K17828	dihydroorotate dehydrogenase (NAD+) catalytic subunit	none	Bacteria	6	0.02241	-4.218
K20108	maltose PTS system EIICB or EIICBA component	phylum	Bacillota	3	0.04898	4.336
K06078	murein lipoprotein	phylum	Pseudomonadota	17	$5.7 \cdot 10^{-7}$	1.800
K03809	NAD(P)H dehydrogenase (quinone)	phylum	Pseudomonadota	6	0.01782	1.713
K03746	DNA-binding protein H-NS	phylum	Pseudomonadota	10	0.03719	1.156
K11685	DNA-binding protein StpA	phylum	Pseudomonadota	9	0.03719	1.150

KO	KO name	taxonomic specificity	taxon name	peptides	q-value	difference (T1-T0)
K15771	arabinogalactan oligomer / maltooligosaccharide transport system permease protein	phylum	Bacillota	17	0.03719	1.052
K11934	outer membrane protein X	phylum	Pseudomonadota	10	0.03719	0.976
K05606	methylmalonyl-CoA/ethylmalonyl-CoA epimerase	phylum	Bacillota	33	0.01203	0.955
K00656	formate C-acetyltransferase	phylum	Pseudomonadota	34	0.02753	0.937
K01761	methionine-gamma-lyase	phylum	Bacillota	15	0.03719	0.921
K16076	outer membrane porin protein LC	phylum	Pseudomonadota	84	0.04898	0.873
K11929	outer membrane pore protein E	phylum	Pseudomonadota	114	0.02812	0.858
K09475	outer membrane pore protein C	phylum	Pseudomonadota	108	0.03249	0.851
K00005	glycerol dehydrogenase	phylum	Pseudomonadota	23	0.03822	0.833
K00096	glycerol-1-phosphate dehydrogenase [NAD(P)+]	phylum	Pseudomonadota	23	0.03822	0.833
K15633	2,3-bisphosphoglycerate-independent phosphoglycerate mutase	phylum	Bacteroidota	30	0.03822	0.796
K01810	glucose-6-phosphate isomerase	phylum	Bacillota	110	0.03249	0.790
K09476	outer membrane pore protein F	phylum	Pseudomonadota	124	0.04036	0.773
K14062	outer membrane protein N	phylum	Pseudomonadota	124	0.04036	0.773
K00821	acetylornithine/N-succinyldiaminopimelate aminotransferase	phylum	Bacillota	45	0.03719	0.746
K03286	OmpA-OmpF porin, OOP family	phylum	Pseudomonadota	111	0.03719	0.586
K01804	L-arabinose isomerase	phylum	Bacteroidota	91	0.01333	-0.686
K06334	spore coat protein JC	phylum	Bacillota	36	0.03719	-0.800
K13963	serpin B	phylum	Bacteroidota	97	0.02263	-0.806
K02110	F-type H+-transporting ATPase subunit c	phylum	Bacillota	30	0.03249	-1.022
K02777	sugar PTS system EIIA component	class	Bacilli	3	0.04934	4.336
K20108	maltose PTS system EIICB or EIICBA component	class	Bacilli	3	0.04934	4.336
K05878	phosphoenolpyruvate---glycerone phosphotransferase subunit DhaK	class	Gammaproteobacteria	6	0.03794	3.787
K03809	NAD(P)H dehydrogenase (quinone)	class	Gammaproteobacteria	5	0.02235	1.705
K03746	DNA-binding protein H-NS	class	Gammaproteobacteria	10	0.03747	1.156
K11685	DNA-binding protein StpA	class	Gammaproteobacteria	9	0.03749	1.150
K00262	glutamate dehydrogenase (NADP+)	class	Bacilli	9	0.02242	1.132
K15771	arabinogalactan oligomer / maltooligosaccharide transport system permease protein	class	Bacilli	16	0.03891	1.022
K01689	enolase	class	Bacilli	61	0.04249	1.001
K11934	outer membrane protein X	class	Gammaproteobacteria	10	0.03749	0.976
K05606	methylmalonyl-CoA/ethylmalonyl-CoA epimerase	class	Negativicutes	33	0.01317	0.955
K00656	formate C-acetyltransferase	class	Gammaproteobacteria	32	0.03747	0.955
K00005	glycerol dehydrogenase	class	Gammaproteobacteria	23	0.03794	0.833
K00096	glycerol-1-phosphate dehydrogenase [NAD(P)+]	class	Gammaproteobacteria	23	0.03794	0.833
K15633	2,3-bisphosphoglycerate-independent phosphoglycerate mutase	class	Bacteroidia	30	0.03794	0.796
K10254	oleate hydratase	class	Clostridia	41	0.03747	0.790

KO	KO name	taxonomic specificity	taxon name	peptides	q-value	difference (T1-T0)
K00821	acetylornithine/N-succinyldiaminopimelate aminotransferase	class	Clostridia	42	0.03747	0.772
K03286	OmpA-OmpF porin, OOP family	class	Gammaproteobacteria	78	0.04841	0.643
K01804	L-arabinose isomerase	class	Bacteroidia	89	0.02235	-0.661
K06334	spore coat protein JC	class	Clostridia	36	0.03747	-0.800
K13963	serpin B	class	Bacteroidia	97	0.02242	-0.806
K02110	F-type H+-transporting ATPase subunit c	class	Clostridia	26	0.03526	-1.067
K05878	phosphoenolpyruvate---glycerone phosphotransferase subunit DhaK	order	Enterobacterales	5	0.03032	3.945
K03746	DNA-binding protein H-NS	order	Enterobacterales	9	0.02768	1.261
K11685	DNA-binding protein StpA	order	Enterobacterales	8	0.03032	1.260
K03286	OmpA-OmpF porin, OOP family	order	Enterobacterales	35	$7.9 \cdot 10^{-5}$	1.154
K00262	glutamate dehydrogenase (NADP+)	order	Lactobacillales	9	0.02097	1.132
K15771	arabinogalactan oligomer / maltooligosaccharide transport system permease protein	order	Lactobacillales	16	0.04278	1.022
K11934	outer membrane protein X	order	Enterobacterales	10	0.03944	0.976
K00134	glyceraldehyde 3-phosphate dehydrogenase (phosphorylating)	order	Lactobacillales	77	0.00927	0.962
K00005	glycerol dehydrogenase	order	Enterobacterales	19	0.03124	0.919
K00096	glycerol-1-phosphate dehydrogenase [NAD(P)+]	order	Enterobacterales	19	0.03124	0.919
K15633	2,3-bisphosphoglycerate-independent phosphoglycerate mutase	order	Bacteroidales	30	0.04187	0.796
K10254	oleate hydratase	order	Eubacteriales	41	0.03506	0.790
K00821	acetylornithine/N-succinyldiaminopimelate aminotransferase	order	Eubacteriales	42	0.03248	0.772
K00626	acetyl-CoA C-acetyltransferase	order	Eubacteriales	175	0.02016	0.481
K01804	L-arabinose isomerase	order	Bacteroidales	86	0.01806	-0.666
K06334	spore coat protein JC	order	Eubacteriales	36	0.03624	-0.800
K13963	serpin B	order	Bacteroidales	97	0.02097	-0.806
K02110	F-type H+-transporting ATPase subunit c	order	Eubacteriales	26	0.03032	-1.067
K01624	fructose-bisphosphate aldolase, class II	family	Streptococcaceae	6	0.01435	1.294
K03339	6-phospho-5-dehydro-2-deoxy-D-gluconate aldolase	family	Streptococcaceae	6	0.01435	1.294
K00134	glyceraldehyde 3-phosphate dehydrogenase (phosphorylating)	family	Streptococcaceae	31	0.00131	1.256
K00262	glutamate dehydrogenase (NADP+)	family	Streptococcaceae	9	0.02035	1.132
K00794	6,7-dimethyl-8-ribityllumazine synthase	family	Prevotellaceae	10	0.04103	1.007
K00873	pyruvate kinase	family	Streptococcaceae	27	0.00640	0.939
K01689	enolase	genus	Streptococcus	17	0.04017	1.339
K01624	fructose-bisphosphate aldolase, class II	genus	Streptococcus	6	0.01261	1.294
K03339	6-phospho-5-dehydro-2-deoxy-D-gluconate aldolase	genus	Streptococcus	6	0.01261	1.294
K00134	glyceraldehyde 3-phosphate dehydrogenase (phosphorylating)	genus	Streptococcus	30	0.00115	1.255
K00262	glutamate dehydrogenase (NADP+)	genus	Streptococcus	9	0.01823	1.132

KO	KO name	taxonomic specificity	taxon name	peptides	q-value	difference (T1-T0)
K15633	2,3-bisphosphoglycerate-independent phosphoglycerate mutase	genus	Prevotella	17	0.04289	1.018
K00794	6,7-dimethyl-8-ribityllumazine synthase	genus	Prevotella	10	0.03519	1.007
K01119	2',3'-cyclic-nucleotide 2'-phosphodiesterase / 3'-nucleotidase	genus	Faecalibacterium	13	0.04064	0.990
K00873	pyruvate kinase	genus	Streptococcus	25	0.00848	0.961
K03640	peptidoglycan-associated lipoprotein	genus	Desulfovibrio	10	0.00848	0.877
K13963	serpin B	genus	Prevotella	78	0.04353	-0.788

Table S4. Human proteins with significantly higher abundance in T1 compared to T0. Difference values and q-values were obtained by paired sample Student's t-test followed by Benjamini and Hochberg's adjustment for multiple testing. Proteins are ordered by decreasing difference value.

UniProt accession	Protein name	STRING cluster	Enriched Reactome pathways	q-value	Difference (T1-T0)
Q15365	Poly(rC)-binding protein 1	3 (glycolytic process)		0.003939	7.107
P04040	Catalase		Immune System | Neutrophil degranulation	0.018250	4.576
P07686	Beta-hexosaminidase subunit beta		Immune System | Neutrophil degranulation	0.025461	4.073
P23528	Cofilin-1	14	Hemostasis | Immune System	0.043263	3.738
Q00325	Phosphate carrier protein, mitochondrial	5		0.029113	2.544
P09972	Fructose-bisphosphate aldolase C	3 (glycolytic process)	Glycolysis | Immune System | Neutrophil degranulation	0.028664	2.507
P12273	Prolactin-inducible protein	9 (miscellaneous transport and binding)		0.003939	2.423
P34931	Heat shock 70 kDa protein 1-like	13 (attenuation phase)	Immune System	0.049740	2.330
P16422	Epithelial cell adhesion molecule		Hemostasis	0.041264	2.271
P08123	Collagen alpha-2(I) chain	10 (anchoring fibril formation)	Hemostasis | Immune System	0.018250	1.811
P00338	L-lactate dehydrogenase A chain	3 (glycolytic process)	Aerobic respiration and respiratory electron transport	0.002121	1.661
P31025	Lipocalin-1	1 (defense response to fungus)		0.032028	1.602
P01857	Immunoglobulin heavy constant gamma 1		Immune System	0.003584	1.595
P08572	Collagen alpha-2(IV) chain	10 (anchoring fibril formation)		0.023935	1.443
O43790	Keratin, type II cuticular Hb6			0.036997	1.430
P48735	Isocitrate dehydrogenase [NADP], mitochondrial	4 (citrate cycle)	Aerobic respiration and respiratory electron transport	0.002251	1.373
P02788	Lactotransferrin	1 (defense response to fungus)	Antimicrobial peptides | Immune System | Neutrophil degranulation	0.000002	1.366
P04075	Fructose-bisphosphate aldolase A	3 (glycolytic process)	Glycolysis | Hemostasis | Immune System | Neutrophil degranulation	0.002251	1.349
P01023	Alpha-2-macroglobulin	1 (defense response to fungus)	Hemostasis	0.000307	1.258
P00558	Phosphoglycerate kinase 1	3 (glycolytic process)	Glycolysis	0.000146	1.255
P55259	Pancreatic secretory granule membrane major glycoprotein GP2			0.002264	1.234
Q06141	Regenerating islet-derived protein 3-alpha		Antimicrobial peptides | Immune System	0.012390	1.155

UniProt accession	Protein name	STRING cluster	Enriched Reactome pathways	q-value	Difference (T1-T0)
P06748	Nucleophosmin	4 (citrate cycle)	Immune System	0.002251	1.133
P01591	Immunoglobulin J chain	12 (secretory IgA complex)	Hemostasis	0.005315	1.131
P12955	Xaa-Pro dipeptidase			0.023503	1.107
P08311	Cathepsin G	1 (defense response to fungus)	Antimicrobial peptides | Immune System | Neutrophil degranulation	0.000434	1.093
P68133	Actin, alpha skeletal muscle	2 (striated muscle contraction)	Muscle contraction	0.009994	1.065
Q9BYF1	Angiotensin-converting enzyme 2	6 (meprin A complex)		0.003939	1.039
P01911	HLA class II histocompatibility antigen, DRB1 beta chain	7	Immune System	0.015866	1.018
P25705	ATP synthase subunit alpha, mitochondrial	5	Aerobic respiration and respiratory electron transport	0.006789	1.003
P14618	Pyruvate kinase PKM	3 (glycolytic process)	Aerobic respiration and respiratory electron transport | Glycolysis | Immune System | Neutrophil degranulation	0.001277	0.995
P13533	Myosin-6	2 (striated muscle contraction)	Muscle contraction	0.018250	0.994
P17540	Creatine kinase S-type, mitochondrial	8 (phosphocreatine biosynthesis)		0.015224	0.978
P05109	Protein S100-A8	1 (defense response to fungus)	Antimicrobial peptides | Immune System | Neutrophil degranulation	0.015866	0.977
P02768	Albumin	16	Hemostasis	0.002251	0.941
P01024	Complement C3	1 (defense response to fungus)	Immune System | Neutrophil degranulation	0.006288	0.941
P08237	ATP-dependent 6-phosphofructokinase, muscle type	3 (glycolytic process)	Glycolysis	0.002121	0.923
Q7L5L3	Lysophospholipase D GDPD3	11		0.014179	0.920
Q16819	Meprin A subunit alpha	6 (meprin A complex)		0.002251	0.896
P09622	Dihydrolipoyl dehydrogenase, mitochondrial	5	Aerobic respiration and respiratory electron transport	0.032028	0.893
P05164	Myeloperoxidase	1 (defense response to fungus)	Immune System | Neutrophil degranulation	0.002251	0.874
P23396	40S ribosomal protein S3	15		0.043263	0.874
Q05639	Elongation factor 1-alpha 2	7		0.008106	0.858
P40926	Malate dehydrogenase, mitochondrial	4 (citrate cycle)	Aerobic respiration and respiratory electron transport	0.041264	0.855

UniProt accession	Protein name	STRING cluster	Enriched Reactome pathways	q-value	Difference (T1-T0)
P35609	Alpha-actinin-2	2 (striated muscle contraction)	Hemostasis | Muscle contraction	0.002610	0.844
P04054	Phospholipase A2	11		0.017999	0.842
P0DUB6	Alpha-amylase 1A			0.039788	0.834
P01871	Immunoglobulin heavy constant mu		Hemostasis | Immune System	0.003584	0.827
Q9HC84	Mucin-5B	1 (defense response to fungus)	Immune System	0.025655	0.825
P08246	Neutrophil elastase	1 (defense response to fungus)	Antimicrobial peptides | Immune System | Neutrophil degranulation	0.011742	0.812
P63316	Troponin C, slow skeletal and cardiac muscles	2 (striated muscle contraction)	Muscle contraction	0.049456	0.806
Q9Y623	Myosin-4	2 (striated muscle contraction)		0.009440	0.804
Q08380	Galectin-3-binding protein		Hemostasis	0.000197	0.802
P09493	Tropomyosin alpha-1 chain	2 (striated muscle contraction)	Muscle contraction	0.043115	0.788
O14983	Sarcoplasmic/endoplasmic reticulum calcium ATPase 1	2 (striated muscle contraction)	Hemostasis | Muscle contraction	0.012906	0.787
P06732	Creatine kinase M-type	8 (phosphocreatine biosynthesis)		0.042083	0.784
P11142	Heat shock cognate 71 kDa protein	13 (attenuation phase)	Immune System | Neutrophil degranulation	0.009440	0.784
P25311	Zinc-alpha-2-glycoprotein	9 (miscellaneous transport and binding)		0.002121	0.783
P13929	Beta-enolase	3 (glycolytic process)	Glycolysis	0.014993	0.774
P01833	Polymeric immunoglobulin receptor	12 (secretory IgA complex)	Immune System | Neutrophil degranulation	0.003813	0.748
P00390	Glutathione reductase, mitochondrial	4 (citrate cycle)		0.041652	0.719
P07951	Tropomyosin beta chain	2 (striated muscle contraction)	Muscle contraction	0.041264	0.715
P00915	Carbonic anhydrase 1		Immune System	0.043263	0.713
P56470	Galectin-4			0.017999	0.706
P60709	Actin, cytoplasmic 1	14	Hemostasis | Immune System	0.043115	0.693
Q13642	Four and a half LIM domains protein 1			0.049456	0.686
P12883	Myosin-7	2 (striated muscle contraction)		0.041264	0.677
P09668	Pro-cathepsin H		Immune System | Neutrophil degranulation	0.031406	0.673
Q16820	Meprin A subunit beta	6 (meprin A complex)		0.019704	0.664

UniProt accession	Protein name	STRING cluster	Enriched Reactome pathways	q-value	Difference (T1-T0)
P30740	Leukocyte elastase inhibitor	1 (defense response to fungus)	Immune System | Neutrophil degranulation	0.026678	0.650
P01876	Immunoglobulin heavy constant alpha 1		Hemostasis	0.043455	0.624
Q03154	Aminoacylase-1			0.041264	0.597
P08779	Keratin, type I cytoskeletal 16			0.023503	0.582
O43451	Maltase-glucoamylase, intestinal		Immune System | Neutrophil degranulation	0.043263	0.572

Table S5. Anthropometric and blood parameters measured in the patients recruited in this study.

Sample code	Time point	Neck (cm)	Waist (cm)	Hip (cm)	HbA1c (mmol/ml)	Cholesterol (mg/dL)	Glucose (mg/dL)	Triglycerides (mg/dL)	LDL (mg/dL)	HDL (mg/dL)	Cortisol (µg/mL)
G01	T0	36	116	118	34	198	75	96	110	68	92
G02	T0	54	155	150	54	209	95	63	136	61	112
G05	T0	41	104	120	33	163	87	104	87	55	170
G06	T0	35	120	118	35	167	89	82	91	53	125
G09	T0	49	140	130	36	262	90	114	181	59	86
G10	T0	43	127	132	37	192	139	131	129	37	130
G11	T0	48	162	155	43	179	101	163	108	38	112
G12	T0	41	135	125	62	137	153	105	79	36	202
G16	T0	45	130	122	40	152	81	59	87	54	125
G17	T0	53	150	145	93	219	159	103	147	52	86
G19	T0	42	142	127	44	257	102	141	169	61	98
G23	T0	44	144	128	43	214	111	149	142	42	40
G24	T0	48	152	148	39	197	87	67	139	45	57
G28	T0	45	142	140	38	197	78	88	122	57	64
G30	T0	39	112	132	38	246	90	117	173	49	202
G31	T0	42	110	130	35	190	75	96	116	55	73
G32	T0	40	112	132	37	180	85	107	91	62	82
N03	T0	36.5	103	128	35	104	73	23	84	64	10
N04	T0	36	121.5	126	33	159	88	125	99	49	19.1
N07	T0	41	125	138	47	192	111	156	145	37	17.6
N11	T0	49	131	136	44	232	91	119	196	36	132
N13	T0	37	121	127	40	182	96	95	136	39	82
N14	T0	38	104	124	33	170	78	98	90	56	111
N15	T0	41.5	122	139	38	199	85	122	150	33	91
N16	T0	42	135	130	41	202	120	81	132	67	112
N18	T0	37	131	150	37	173	90	95	113	47	83
N19	T0	49	145	130	45	152	81	108	91	46	136
N20	T0	43	117	135	35	265	90	208	208	50	68
N21	T0	42	119	139	37	191	90	142	123	49	31
N24	T0	51	130	140	50	246	100	268	162	52	20
N25	T0	44	123	142	55	217	98	181	173	34	77
N26	T0	48	124	138	32	184	93	83	114	62	112
N27	T0	46	123	128	35	140	92	331	72	27	102
R10	T0	38	130	145	31	167	96	89	120	29	110

Sample code	Time point	Neck (cm)	Waist (cm)	Hip (cm)	HbA1c (mmol/ml)	Cholesterol (mg/dL)	Glucose (mg/dL)	Triglycerides (mg/dL)	LDL (mg/dL)	HDL (mg/dL)	Cortisol (µg/mL)
R11	T0	39	115	134	32	205	100	150	126	49	132
R12	T0	46	149	150	28	149	87	94	84	46	10
R14	T0	39.5	138	129	29	154	89	112	104	27	9.7
R15	T0	37.5	123	136	29	221	89	216	180	36	13
R16	T0	42	125	130	5.3	241	108	212	154	45	12.1
R18	T0	36	101	140	33	146	92	118	84	38	104
R19	T0	33	132	112.5	33	285	100	199	186	59	144
R20	T0	43	120	139	36	203	111	132	170	33	14
R27	T0	37	113	137	36	146	86	102	73	53	73
R32	T0	52	136	133	0	191.25	95	118	181	59	180
R33	T0	45	140	138	53	146	124	154	90	40	8.7
G01	T1	31	78	90	31	156	76	70	100	32	66
G02	T1	47	142	133	32	170	82	124	75	70	125
G05	T1	35	90	116	30	120	88	92	44.6	58	7.2
G06	T1	40	87	101	39	155	64	78	80	39	8.9
G09	T1	40	94	110	33	112	80	75	70	27	11.5
G10	T1	37	87	98	35	131	85	70	85	32	232
G11	T1	43	120	130	41	107	95	95	48	40	13.9
G12	T1	42	105	112	33	120	87	91	67	35	11.7
G16	T1	38	100	110	38	115	75	103	65	30	13
G17	T1	42	114	128	45	120	100	85	77	26	9.4
G19	T1	42	101	110	42	130	65	80	79	35	9.9
G23	T1	36	95	105	32	175	84	80	142	48	13.5
G24	T1	38	100	119	38	115	91	80	70	29	21.5
G28	T1	34	88	120	32	170	83	75	122	33	8.2
G30	T1	34	106	110	34	229	88	112	145	62	55.2
G31	T1	34	82	107	34	162	87	52	89	58	12
G32	T1	31	80	112	34	179	86	76	92	70	11.2
N03	T1	32	79	100	37	126	71	28	37	75	8.9
N04	T1	33	86	111	33	150	75	68	76	59	22.8
N07	T1	34	95	103	35	157	80	59	103	50	23
N11	T1	39	94	100	38	156	83	60	89	57	14.6
N13	T1	32	93	109	40	192	84	102	113	58	11.4
N14	T1	104	104	99	37	130	77	70	50	60	8.1
N15	T1	36	102	115	36	187	78	114	106.2	58	12.4
N16	T1	36	96	120	37	171	83	85	92	62	15.8

Sample code	Time point	Neck (cm)	Waist (cm)	Hip (cm)	HbA1c (mmol/ml)	Cholesterol (mg/dL)	Glucose (mg/dL)	Triglycerides (mg/dL)	LDL (mg/dL)	HDL (mg/dL)	Cortisol (µg/mL)
N18	T1	34	102	125	37	138	69	63	78	57	6
N19	T1	36	89	97	35	116	72	51	56	56	12
N20	T1	32.5	87	101	32	233	72	65	156	68	12
N21	T1	35.5	83	110	34	168	71	67	107	66	6.1
N24	T1	38.5	105	120	40	190	86	52	140	61	6.6
N25	T1	42	107	107	30	172	80	76	126	43	22.1
N26	T1	40	104.5	113	28	153	78	69	93	61	10.4
N27	T1	39	100	106	35	126	73	143	80	32	6.2
R10	T1	34	83	86	26	151	83	53	92.4	48	13.2
R11	T1	35	95	105	25	195	87	132	95	73	131
R12	T1	36	80	95	26	142	83	63	76.4	53	104
R14	T1	32	110	99	25	135	79	79	110.8	40	61
R15	T1	34.5	98.5	106.3	19	163	53	59	78.2	73	101
R16	T1	37	36	103	29	220	90	119	135.2	61	12.4
R18	T1	31.5	72	91.4	25	117	81	62	52.6	52	85
R19	T1	34	105	116	28	184	87.1	122	120.6	39	156
R20	T1	39	113	131	33	202	86	110	135	45	119
R27	T1	35.2	92.5	102	28	143	88	106	110	54	18.2
R32	T1	38	93	108	31	145	96	212	148.4	39	45.2
R33	T1	37	102	100	25	123	85	65	54	56	87.9
